# Fissure locations and fire-fountain dynamics during the December 2023–September 2024 Svartsengi Volcanic System eruptions, Iceland, from aerial imagery and recreational webcam footage

**DOI:** 10.1007/s00445-026-02006-3

**Published:** 2026-07-11

**Authors:** Rebekah S. Rhodes, David M. Pyle, Tamsin A. Mather, Gro B. M. Pedersen, Michelle M. Parks

**Affiliations:** 1 Department of Earth Sciences, University of Oxford, Oxford, OX1 3AN UK; 2 The Icelandic Meteorological Office, Reykjavík, Iceland

**Keywords:** Fissure eruptions, Fire-fountaining, Svartsengi Volcanic System, Video analysis, Reykjanes Peninsula

## Abstract

Recent fissure-fed eruptions from the Svartsengi Volcanic System have significantly impacted Iceland’s Reykjanes Peninsula. With eruptions likely to continue intermittently for decades to centuries, exploring the controls on the locations of fissure-fed eruptions is important for hazard assessment and risk mitigation. This study maps eruptive fissures from six eruptions from December 2023 to September 2024 using satellite and aerial imagery and observes fire-fountain height evolution for four eruptions using webcam footage. We find that fissure locations are heavily influenced by topography, including features parallel to fissure strike and features formed during previous eruptions. The maximum fire-fountain heights ranged from 56 m during the January 2024 eruption to 133 m during the August 2024 eruption. Variations in fire-fountain heights in a single eruption between different fissure segments, and through time, appear to primarily be controlled by surface vent size and dike pressurisation, both of which increase mass eruption rate and lead to higher fountains. Understanding the controls on fissure location highlights areas most vulnerable to future fissure opening, which can help inform ongoing hazard assessment and risk mitigation. Digital video capture of future eruptions from multiple angles would augment monitoring and help refine models of fire-fountain evolution.

**Supplementary Information:**

The online version contains supplementary material available at 10.1007/s00445-026-02006-3.

## Introduction

**Fig. 1:**
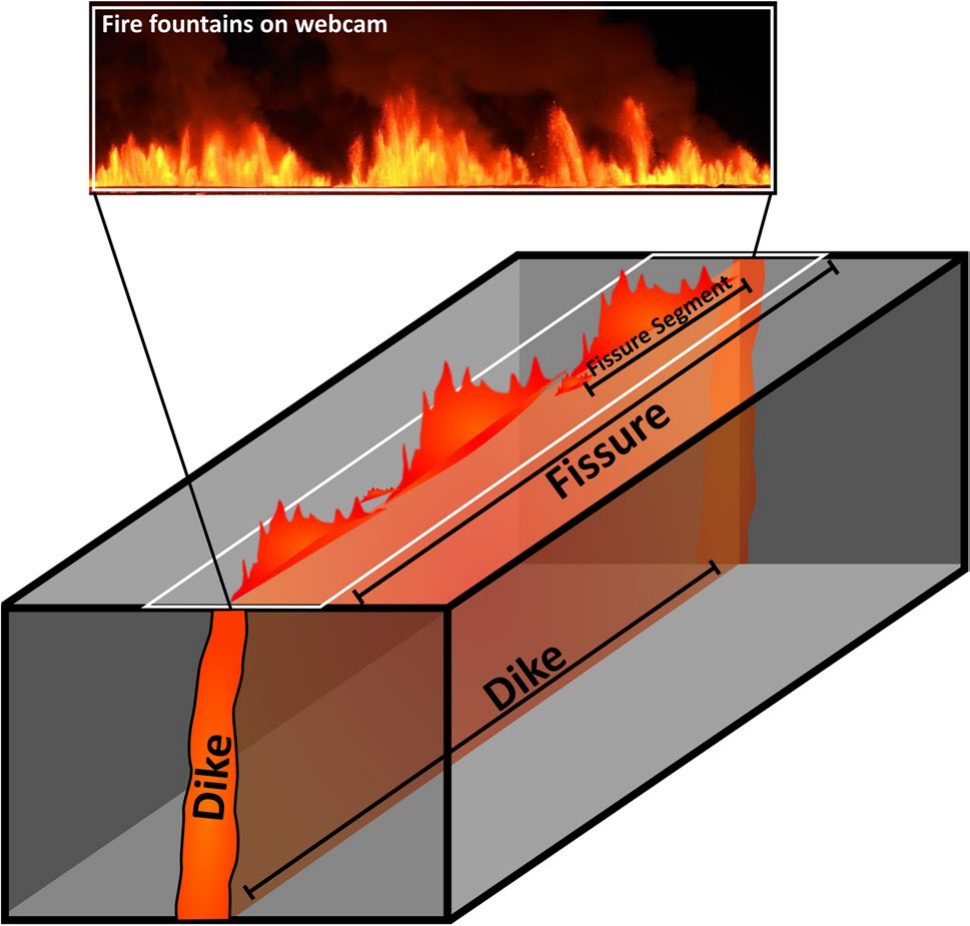
Schematic of the relationship between a dike and a surface fissure, which is broken into fissure segments. The photograph shows how the fissure and fire-fountains appear on the webcam footage. Dikes in the Svartsengi Volcanic System are thought to be steeply dipping to vertical, and range in length from 2.5 km (January 2024) to 15 km (November 2023). Fissure lengths range from < 1 km during the January 2024 eruption to 5.5 km in the August 2024 eruption (Parks et al. 2025)

Fissure-fed fire-fountains form when mafic magma erupts from laterally extensive dikes (Fig. 1). Dikes propagate until they arrest or intersect the surface in an eruption. From this intersection point with the surface, eruptive fissures propagate to form a series of discontinuous fissure segments from which magma erupts (Tyrrell, 1937; Hurley et al. 2025). During an eruption, some fissure segments deactivate with activity concentrating into one or a few cones until the eruption ceases (Wylie et al. 1999). In ideal conditions and homogeneous material, dikes and their associated fissures propagate perpendicular to the direction of least compressive stress (σ3; Anderson 1951). However, in real-world conditions where subsurface material is heterogeneous and stress conditions are nonuniform, many factors influence the propagation paths of dikes and fissures, including preexisting crustal weaknesses (Delaney et al. 1986; Ziv et al. 2000), crustal loading (Klügel et al. 2005; Walter and Amelung 2006; Jenness and Clifton 2009; Hurley et al. 2025), and topography (Gaffney and Damjanac 2006; Hurley et al. 2025).

Fire-fountains are jets or sprays of magma droplets, carried in gas, that discharge from vents or fissures (Calvari et al. 2018; Fig. 1). The heights of fire-fountains are considered to be controlled by factors including magma gas content, mass eruption rate, magma viscosity, surface vent size, and source overpressure (e.g., Wilson and Head 1981; Parfitt et al. 1995). Exsolved volatile content has traditionally been considered the primary control on fire-fountain height (Head and Wilson 1987; Moyer and Sahagian 2024). However, most previous studies have examined single-vent eruptions rather than fissure eruptions (e.g. Wolfe et al. 1987). Among studies of fissure eruptions, many have not focused on the initial stages of eruption, but instead on the period after activity became localised into cones (e.g. Witt et al. 2018). During fissure eruptions, fire-fountain heights vary between fissure segments despite being fed by a single dike. This height variation has remained largely unstudied.

Fissure-fed eruptions can have long spatial and temporal extents, with the potential to significantly impact communities and ecosystems. Understanding where fissures may propagate is important for hazard management and risk mitigation, and aids predictions of where the longest lava flows may form. This can assist the planning and installation of protective measures like berms and barriers (e.g., Barsotti et al. 2023).

**Fig. 2:**
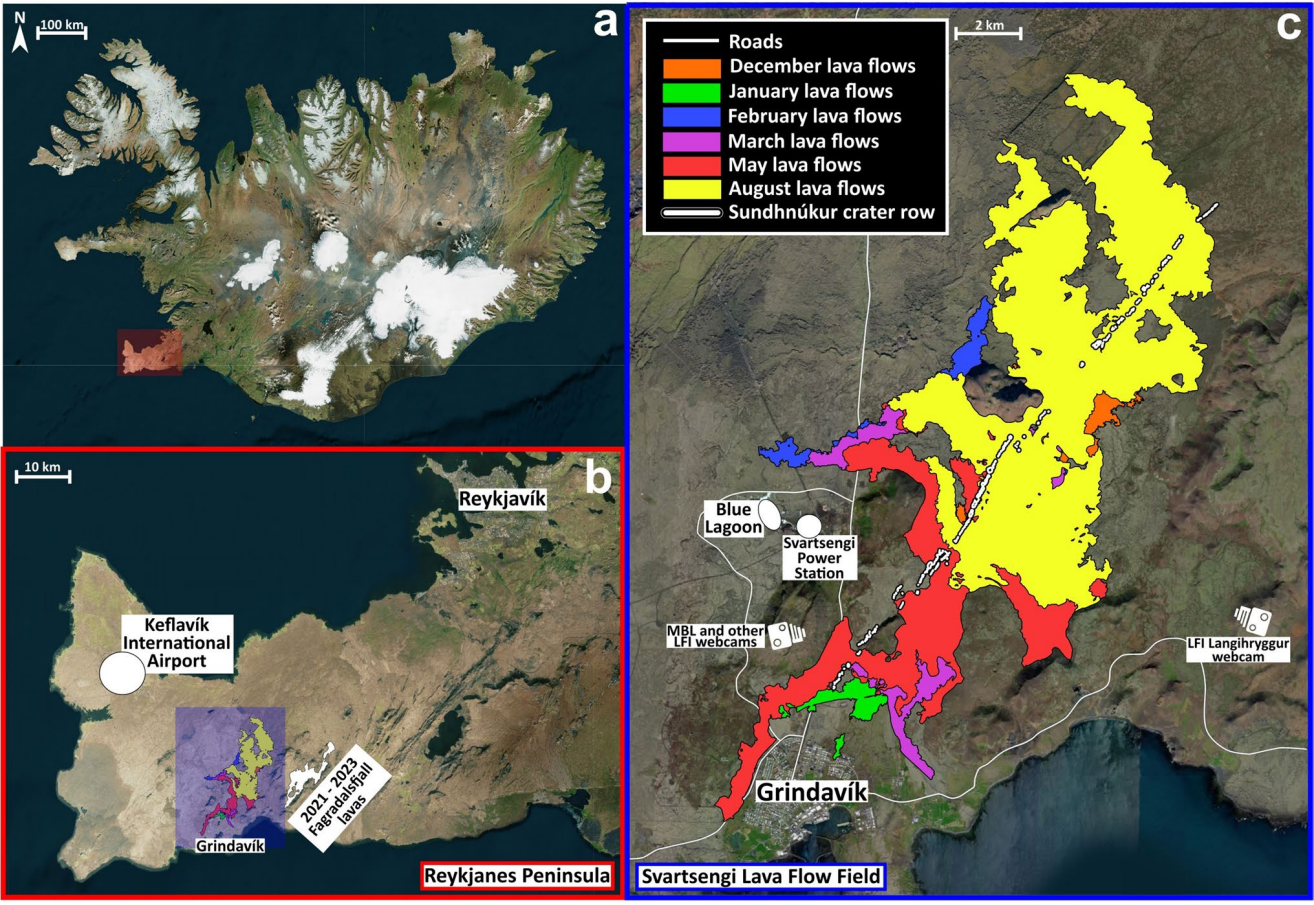
Geographical context of the Reykjanes Peninsula and the Svartsengi lava field. (a) Location of the Reykjanes Peninsula shown in red. (b) Location of Svartsengi lava flow field (blue box) on the Reykjanes Peninsula, and locations of the Fagradalsfjall lava flow field, Keflavík Airport and Reykjavík. (c) Svartsengi lava flow field showing the outlines of lava flows from the first six eruptions of the Svartsengi Volcanic System (December 2023–September 2024). Locations of the webcams used to analyse the eruptions are shown. Key infrastructure is also shown: Grindavík, Svartsengi Power Station, Blue Lagoon, and roads. Base map: Bing (2025). Maps made in QGIS and edited in Inkscape

In December 2019, the Reykjanes Peninsula, Iceland, entered a new period of unrest characterised by magma reservoir inflation and fissure-fed eruptions. The first eruption occurred at Fagradalsfjall in March 2021 (Fig. 2). The peninsula and surrounding region are home to 70% of Iceland’s population and host significant infrastructure and tourism assets, including road links, hot water networks, the Blue Lagoon, and the Svartsengi Power Station (Barsotti et al. 2023; Fig. 2). Evidence from the previous eruptive episode (800–1240 AD) indicates that lava flows reached areas that are now inhabited. Historical records and lava-flow ages suggest that volcanism may continue intermittently for the next few centuries (Caracciolo et al. 2023; Sæmundsson et al. 2020), with the potential for sustained impacts on local communities. Eruptions have already resulted in repeated evacuations from Grindavík and the destruction of property.

Current hypotheses for the factors controlling fire-fountain heights and fissure placement have been developed from examples across different tectonic settings, including Kīlauea (Head and Wilson 1987), Mount Etna (Spampinato et al. 2008), and Holuhraun (Witt et al. 2018). These examples were often stand-alone events, making it challenging to establish the relative importance of each controlling factor generally, rather than for a single event. The repeated eruptions from the Svartsengi Volcanic System provide a natural laboratory to explore these controlling factors without the significant changes in crustal or tectonic conditions that make global comparisons more challenging.

Few fissure eruptions have been documented by video footage spanning their full duration. Most datasets consist either of handheld recordings (e.g., Spampinato et al. 2008: 2002–2003 Mt. Etna eruption), or short clips lasting only a few minutes (e.g., Witt & Walter 2017: March 2011 Pu’u’ō’ō eruption, Kīlauea Volcano). Several studies have examined fire-fountain dynamics during the later stages of eruptions (e.g., Witt et al. 2018: Holuhraun 2014–2015; Scott et al. 2023: Fagradalsfjall 2021; Tisdale et al. 2025: Fagradalsfjall 2022), but observations from the earliest stages of fissure eruptions remain limited. One exception is the 2022 Mauna Loa eruption, for which continuous webcam footage captured fire-fountain activity shortly after eruption onset (Pasqualon et al. 2025), although the first seven minutes were obscured by volcanic fumes.

This study uses recreational webcam footage recorded and live-streamed by several organisations, spanning the full duration of each eruption. We analyse footage from the first six eruptions of the Svartsengi Volcanic System, between December 2023 and September 2024, to explore variations in fire-fountain height from eruption onset, assess the spatial distribution of fissure segments, and evaluate short-term indicators of imminent eruption and rupture location.

## Geological background

### Tectonic and volcanic setting of the Reykjanes Peninsula

Active rifting has occurred on the Reykjanes Peninsula (Fig. 2) for the last 6–7 Ma, generating repeated fissure eruptions (Pałgan et al. 2017; Jóhannesson 1980). Here, the plate boundary is oblique to the direction of plate motion and is divided into six en-echelon volcanic systems (Einarsson 2008; Sæmundsson et al. 2020). Within a 5–10 km wide volcanic and seismic zone, plate motion is accommodated by strike-slip and normal faulting, while volcanic systems are supplied by dikes and erupt along extensional fissures (DeMets et al. 1994; Einarsson 1991; Sigmundsson et al. 2022; Ducrocq et al. 2024). Since 2021, eruptions have occurred in two of these systems, Svartsengi and Fagradalsfjall.

### Historic unrest on the Reykjanes Peninsula

Evidence from historical sources and geochemical data indicates that the last volcanic episode on the Reykjanes Peninsula occurred between 800 and 1240 AD and involved multiple volcanic systems (Sæmundsson et al. 2010; Caracciolo et al. 2023). The dating of lava flows, fissure swarms and tephra layers from four distinct eruptive episodes has revealed that volcanic events on the Reykjanes Peninsula occur in episodic cycles roughly every 1000 years (Einarsson 1991; Klein et al. 1973, 1977; Sigurgeirsson 1992; Sæmundsson and Jóhannesson 2006; Sæmundsson et al. 2020). During volcanic episodes, evidence suggests activity occurs within individual volcanic systems in sequence rather than simultaneously, migrating between volcanic systems as extensional strain shifts along the peninsula (Einarsson 1991; Klein et al. 1977). Since the last period of early post-glacial shield building, volcanism on the Reykjanes Peninsula has typically been characterised by the formation of linear fissures and crater rows (Pedersen and Grosse 2014; Fig. 7).

### Ongoing unrest on the Reykjanes Peninsula

The first fissure eruption of the new volcanic episode on the Reykjanes Peninsula began in March 2021 at Fagradalsfjall. After two years of intermittent unrest and three eruptions, volcanic activity shifted to the Svartsengi volcanic system, which first erupted in December 2023. The eruptions from the Svartsengi Volcanic System are closely spatially associated with the Sundhnúkur crater row, which last erupted approximately 2350 years ago during the Sundhnúkur fires (Jónsson 1983).

**Fig. 3:**
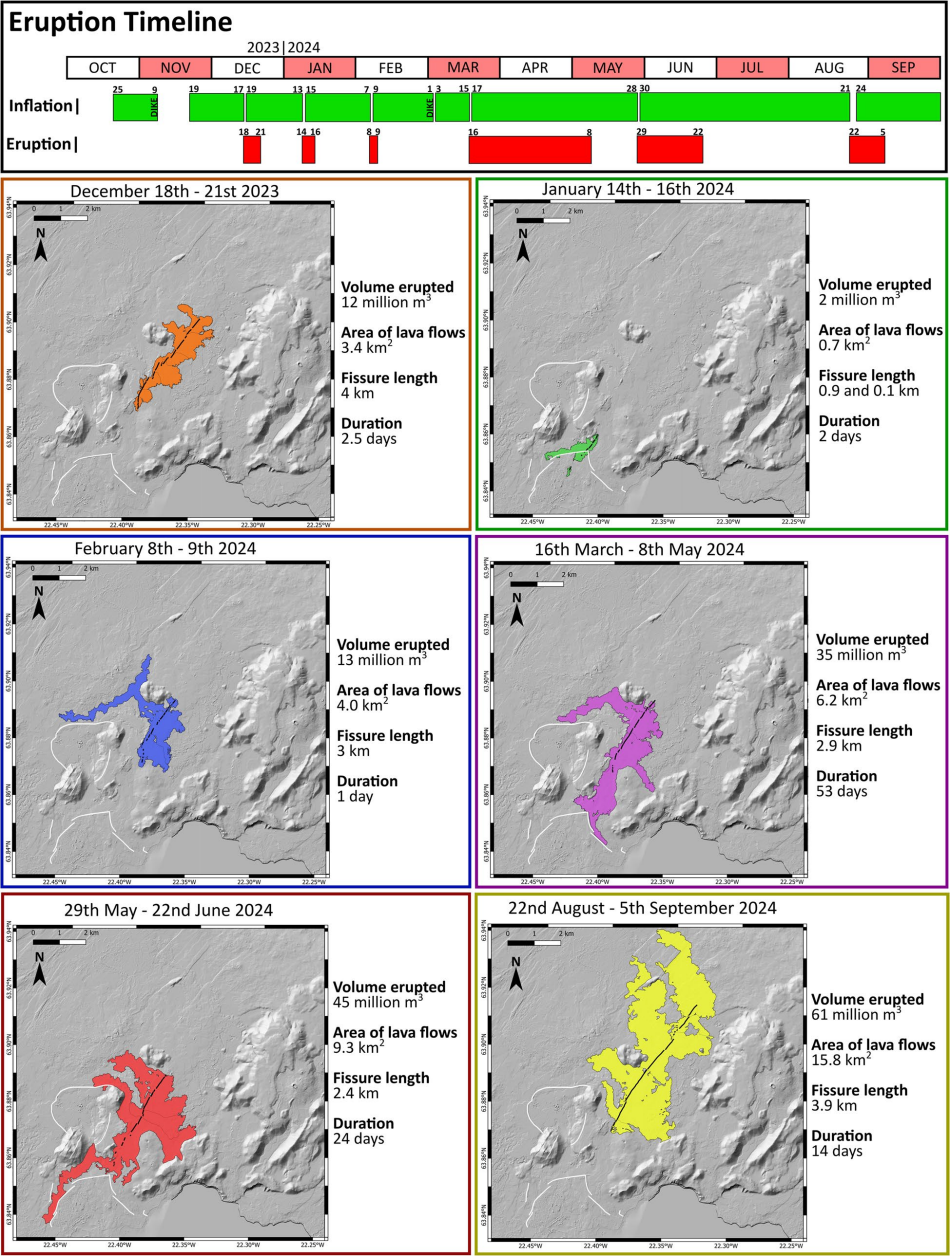
2023–2024 eruption timeline and lava flow outlines for each studied eruption and their associated fissures. Green bars represent the period of magma system inflation, and red bars represent the duration of eruption events. Start and end dates of the inflation and eruption events are shown at the top of both ends of the bars. Zoomed-in versions of these insets with labelled fissure segments can be found in Fig. S1. Protective barriers are shown in white. Base map DEMs from: ÍslandsDEM (2025). Maps made in QGIS and edited on Inkscape. Lava flow outlines: Júlíusdóttir et al. (2024). Eruptions follow the same colour code as Fig. 2

After five separate inflation episodes of the Svartsengi Volcanic System from 2020 to 2023, a sub-vertical dike intruded beneath the Sundhnúkur crater row on 10 November 2023, followed by the first fissure eruption on 18 December 2023 (Geirsson et al. 2021; Cubuk-Sabuncu et al. 2021; Flóvenz et al. 2022; Parks et al. 2023; Sigmundsson et al. 2024). Eleven diking events and nine eruptions occurred between November 2023 and June 2026. Here we focus on the first six eruptions, for which high-quality, continuous webcam footage is available (Fig. 3).

## Data and methods

### Fissure maps

**Fig. 4:**
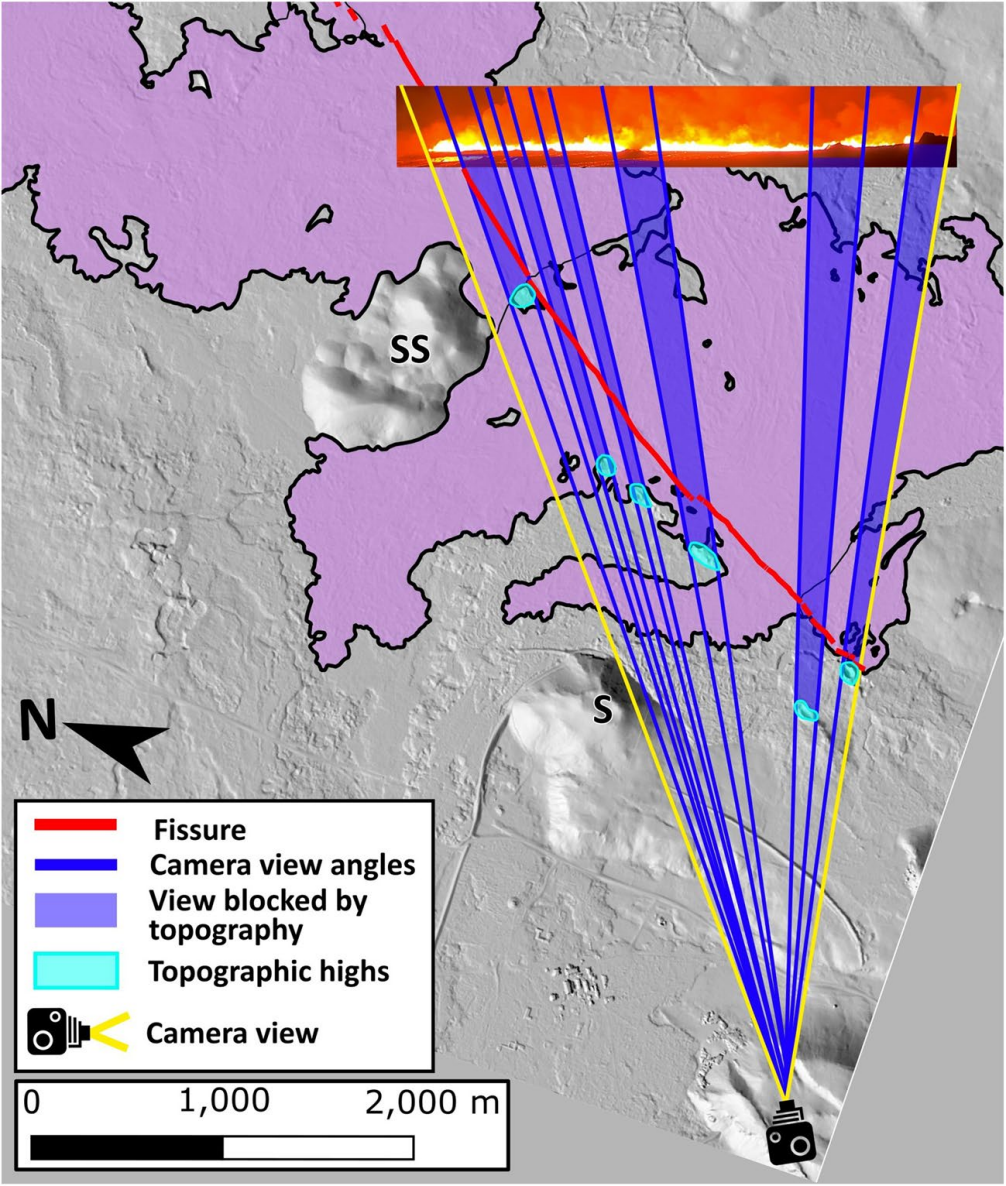
Illustrated method for geolocating webcam fissures. The image shows what the camera sees, which is a projection across the field of view, oblique to the fissure. Topographic highs seen in the webcam footage were matched with the DEM to geolocate the webcam footage and match fissure segments from the footage to the map, accounting for the apparent length of fissures due to the camera angle. This image was captured on 22 August 2024 at 22:34:54. The base map DEM is sourced from ÍslandsDEM (2025). SS = Stóra Skógafell; S = Sylingarfell

Eruptive fissures were mapped at 1–5 m spatial accuracy during and after each eruption using satellite and aerial images, including ICEYE amplitude images, Pleiades images, orthomosaics, digital elevation models (DEMs), and lava thickness maps from flight and drone surveys, with spatial resolution ranging from sub-meter to 3 m per pixel. Fissure traces were mapped in GIS software and refined using helicopter photographs acquired at eruption onset, particularly where fissures were partially buried by later lava flows. The resulting maps were overlain on a DEM to assess topographic controls on fissure location. They were also used to measure fissure segment lengths, geolocate webcam imagery (Fig. 4), and compare recent fissures with those from previous eruptions.

### Fissure segment definition

Fissure segments were defined using QGIS fissure maps, topographic markers (Fig. 4), and webcam footage to identify clear breaks in fissure propagation. These breaks were either temporal, where propagation paused before resuming, or spatial, visible as gaps in the curtain of fire. Once defined, the same segment boundaries were used in both the QGIS fissure maps and the corresponding fire-fountain height time series. Boundaries were not adjusted to reflect changes in fountain behaviour during an eruption. Fissure segments were labelled according to the lava divide they fed and assigned numbers and letters from north to south, rather by opening time (Fig. S1).

### Webcam footage

Our study uses footage from recreational webcams that livestream volcanic activity via public streaming websites. The footage was not recorded for scientific purposes, and the webcams were privately owned and operated by several organisations, including Live from Iceland (LFI), RÚV, Vísir, Morgunblaðið (MBL) and the Icelandic Meteorological Office (IMO). Webcams were located at multiple sites around the Svartsengi Volcanic System and recorded the full duration of each eruption. Here, we analyse footage from the first six eruptions of the Svartsengi Volcanic System (December 2023, January 2024, February 2024, March 2024, May 2024, and August 2024; Fig. 3), comprising 2286 h of footage (table.S5). None of the webcams exhibited barrel distortion.

**Fig. 5:**
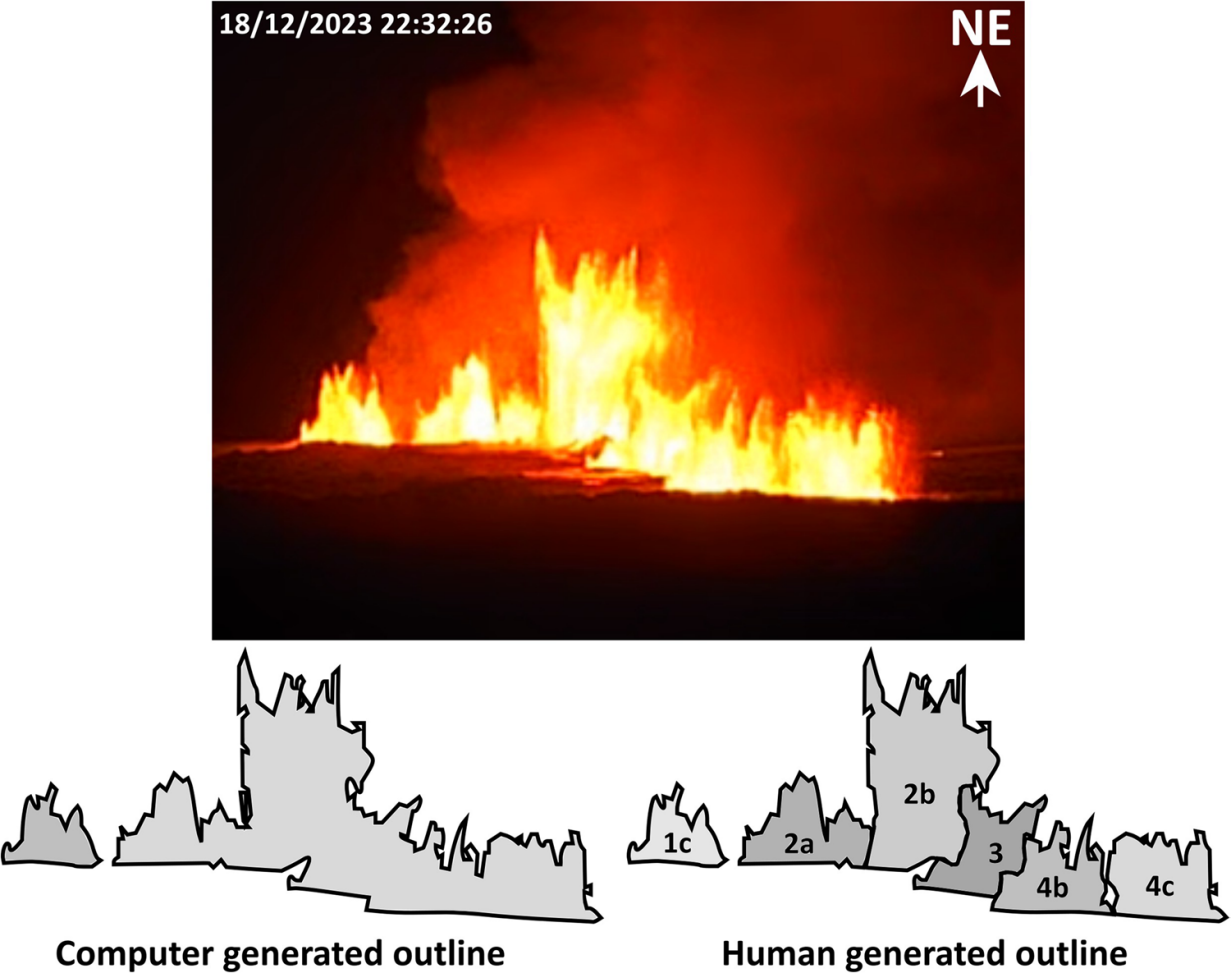
Webcam image still taken during the December 2023 eruption at 22:32:26 from MBL webcam1. The computer-generated outline was made by increasing the contrast in the image and using brightness cutoff software in Inkscape to outline the fire-fountains. From the computer-generated outline, only 2 defined fissure segments are visible. The human-generated outline was made by taking the computer-generated outline and identifying divides between the fissure segments using knowledge from the propagation of the fissure, and the subtle variations in lava spray colour seen in the fire-fountains. From this process, it was possible to identify all the fissure segments present at this time. Segment 4a is missing from this image because it had not propagated yet

Because the webcams were not deployed for scientific monitoring, several limitations affect the analysis. Webcam placement and operation were outside our control, and most cameras were located on Mount Þorbjorn, providing highly oblique views of the eruptive fissures. As the en-echelon fissures propagated, southern segments commonly obscured those farther north. Although individual fissure segments remained visually distinguishable, automated edge-detection and brightness-threshold methods could not reliably separate them, preventing automated collection of segment-specific fire-fountain measurements (Fig. 5). Camera operators also occasionally redirected webcams towards advancing lava flows, interrupting observations of fire-fountains. During the August 2024 eruption, for example, viewing angles were adjusted 2.5 h after eruption onset to follow lava flow emplacement, after which the fountains could no longer be measured. Adverse weather and volcanic fumes also occasionally obscured the fire-fountains.

### Measurements of fire-fountain heights

Footage from the December 2023, January 2024, March 2024, and August 2024 eruptions was of sufficient quality to measure fire-fountain heights and match fissure segments in the footage to mapped segments. Measurements were made for individual fissure segments during the first three hours of each eruption, capturing the initial increase in fountain height and the subsequent decline before activity became concentrated into cones.

Maximum fire-fountain height was measured from the fissure segment to the top of the visible lava spray. Heights were measured directly from webcam footage using a standard ruler held against the monitor, with a measurement uncertainty of ± 1 mm (Fig. S4). On-screen fountain heights ranged from < 1 cm to ~ 20 cm, depending on camera zoom and distance. The on-screen length of each fissure segment was measured in the same way and remeasured whenever the camera zoom or framing changed. All measurements were made using the same monitor, although repeat measurements on different-sized screens and in image-editing software produced near-identical scaled fire-fountain heights.

#### Scaling from screen measurements to real dimensions

To convert on-screen measurements to real-world fire-fountain heights, a scaling factor was calculated for each fissure segment using its mapped length in QGIS.

**Fig. 6:**
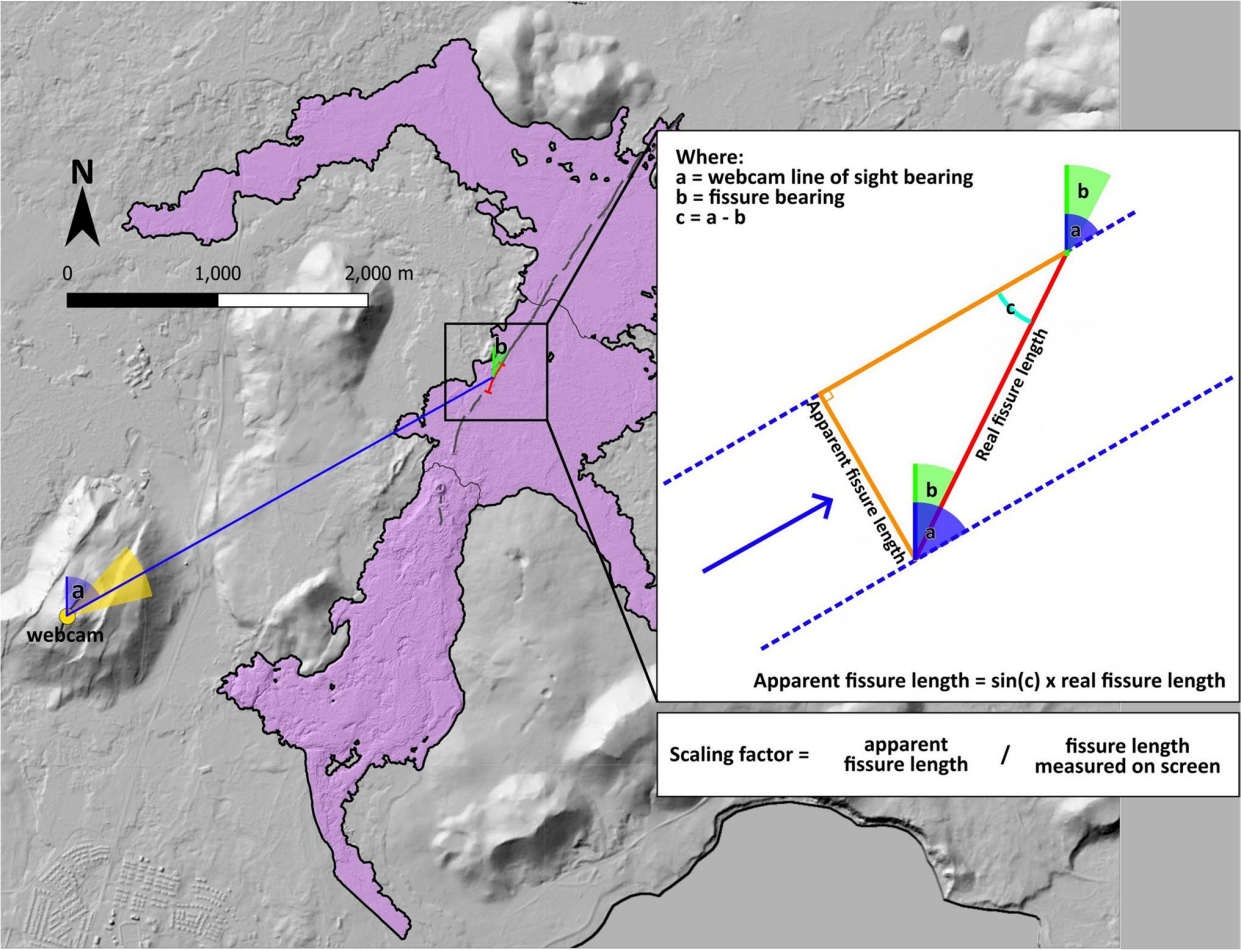
Example calculation of the apparent fissure length seen in the webcam footage of a fissure segment from the March 2024 eruption. This calculation is repeated for every fissure segment, adjusting the fissure bearing and webcam line-of-sight bearing. The scaling factor is then calculated by comparing this calculated apparent fissure segment length to the segment length measured on screen for each different fissure segment. Base map DEM from: ÍslandsDEM (2025)

Because the webcams viewed the fissures obliquely, mapped segment lengths did not correspond directly to their apparent lengths in the footage. To account for this, the mapped segment length, segment bearing, and the bearing of the webcam line of sight to the segment midpoint were used to calculate the segment’s apparent length in real-world units (Fig. 6). A scaling factor was then obtained by comparing this value with the segment’s apparent length measured on-screen and applied to fire-fountain height measurements.

Scaling factors (Eq. (1)) differed between fissure segments because segment orientations and webcam viewing angles varied. In addition, because the webcams were actively operated, scaling factors were recalculated whenever the camera position or zoom changed.

1 $$scaling\mathit\;factor=\frac{\sin\!\;\left(webcam\;line\;of\;sight\;bearing-fissure\;bearing\right)\times real\;scale\;fissure\;length\;(m)}{apparent\;(on\;screen)\;fissure\;length\;(m)}$$

#### Measurement frequency

To capture rapid changes in fire-fountain height during the initial propagation of each fissure segment, measurements were initially taken at 10-s intervals. As rates of change decreased, the sampling interval was progressively increased to 30 s, 1 min, and finally 5 min. Because fountains exhibit strongly pulsatory behaviour, each fissure segment was observed for 10 s at every measurement time, and the maximum fire-fountain height observed was recorded. This approach reduced the measurement variability arising from regular pulsations and enabled consistent comparison between fissure segments and eruptions. Additional measurements were made during notable changes in activity.

#### Uncertainties in fire-fountain heights

Several sources of uncertainty affect the fire-fountain measurements due to the nature of the webcam footage.

1. Measurement uncertainty: Fire-fountain heights and on-screen fissure lengths were measured with a ruler uncertainty of ± 1 mm. Because fissure segment length is used to calculate the scaling factor, its measurement uncertainty introduces uncertainty to the scaling factor. Therefore, larger scaling factors result in greater overall uncertainty (Fig. S2).
2. Webcam location uncertainty: Some webcams were moved between eruptions, and their original location data are no longer available. However, the cameras were not moved significantly, and their approximate positions can be verified using topographic features visible in the footage. To account for the remaining uncertainty, possibility envelopes were used to constrain the range of viewing angles consistent with the inferred camera location (Fig. S3).
3. Fountain-top identification: Identification of the top of a fire fountain requires subjective judgment. This uncertainty is greatest when cameras are zoomed far out, as the lava spray at the top of the fountain may appear as a diffuse glowing cloud rather than a clearly defined boundary.

Measurement and webcam location uncertainties can be quantified. In most cases, measurement uncertainty is the dominant source of error and is shown as error bars on fire-fountain plots. Webcam uncertainty is typically smaller, so the combined measurement and location uncertainty is shown as a grey shaded envelope around the data points.

## Fissure locations

### Results

**Fig. 7:**
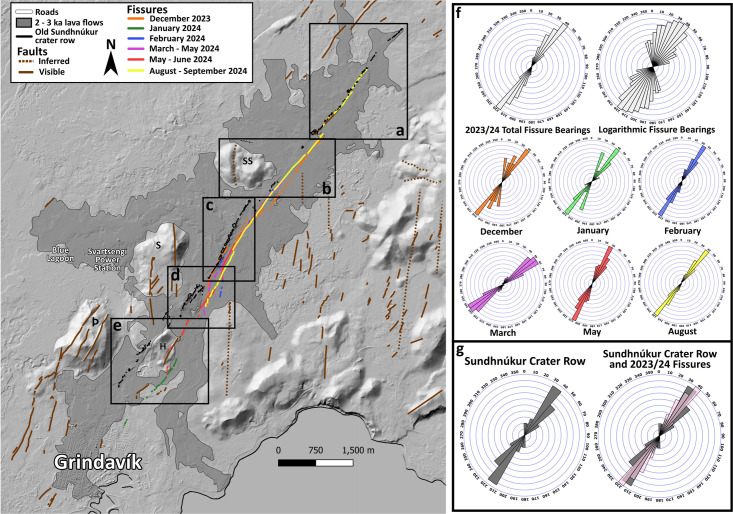
Map of the Svartsengi area, Iceland, showing fissure outlines from the December 2023 to September 2024 eruptions of the Svartsengi Volcanic System, inferred and visible faults in the area and the outline of the old Sundhnúkur crater row. Also shown for reference is the outline of the lava erupted from the Sundhnúkur eruptions at ca. 2350 B.P. GV = Grindavík; Þ = Þorbjorn; H = Hagafell; SV = Sýlingarfell; SS = Stóra Skógafell. Faults, Sundhnúkur crater row outline and Sundhnúkur lava outline from Jenness and Clifton (2009). Base map DEM from: ÍslandsDEM (2025). Map made in QGIS and edited in Inkscape. (a) Northeast area of the fissure field hosting August 2024 fissures that overlap the old crater row. (b) East of Stóra Skógafell fissures are positioned more eastward than the August 2024 fissures in the north. (c) Between Stóra Skógafell and Sýlingarfell. Most common fissure opening location. Most fissures are east of the old crater row and do not overlap fissures from previous eruptions. (d) South of Sýlingarfell. Fissures change strike gradually, avoiding a topographic high made up of Sundhnúkur craters (e) The proposed Hagafell zone of weakness, and topographic deflection of fissures. (f) Rose diagrams of the orientations of fissures during the 2023–2024 eruptions of the Svartsengi Volcanic System. The bearings of each fissure segment have been measured for each eruption and plotted on the Rose diagram. Segments of the Rose diagram are proportional in length to the cumulative length of fissure segments with an orientation in that 10° angle bracket. Total fissure bearings are also shown on a logarithmic plot, so the range of fissure orientations is clearer to see. (g) Rose diagrams of the orientations of fissures of the old Sundhnúkur crater row (left) and the old Sundhnúkur crater row with the total new fissure orientations overlaid in pink (right). Segments are proportional in length to the lengths of fissures

Mapped fissures associated with the December 2023–September 2024 Svartsengi Volcanic System eruptions are shown in Fig. 7, alongside the Sundhnúkur crater row and faults mapped by Jenness and Clifton (2009). Total fissure length ranged from 1 km in the January 2024 eruption to 5.5 km in the August 2024 eruption. These fissures comprise discrete segments that vary both in length, from less than a meter to hundreds of meters, and strike, from N–S to ENE-WSW (Fig. 7f). In homogenous crust, fissure segments would be expected to share a common orientation and lie directly above the underlying dike. Instead, the Svartsengi fissure field exhibits substantial variation in fissure geometry along its length.

In the north-east of the fissure field, there are only fissures that opened during the August 2024 eruption. These fissures are well aligned NE–SW and overlap both the old Sundhnúkur crater row and a normal fault (Fig. 7a).

Further south, fissures that opened next to Stóra Skógafell are positioned more eastward than the August 2024 fissures in the north (Fig. 7b). The fissures do not overlap the old Sundhnúkur crater row in this area.

Between Stóra Skógafell and Sýlingarfell, there is a high density of fissure segments (Fig. 7c). This was the most common fissure opening location during these eruptions. The fissures in this area are generally positioned eastward of the Sundhnúkur crater row. In the north of this section of the crater row, the fissures are positioned up to 335 m to the east of the Sundhnúkur crater row, and at the southern end, by up to 165 m eastward of the Sundhnúkur crater row. Only the December 2023 eruptions had fissure segments that partially overlapped the Sundhnúkur crater row at its southern end. In this area, fissures opening in subsequent eruptions did not perfectly overlap fissures that opened in previous eruptions.

South of Sýlingarfell, fissures change strike gradually, avoiding a topographic high made up of Sundhnúkur craters (Fig. 7d). Similar patterns are observed around Hagafell, where fissures from January 2024 avoid a topographic high. However, during the May 2024 eruption, fissures exploited a normal fault through Hagafell (Fig. 7e).

The southernmost area of the Svartsengi fissure field only hosts fissures from the January 2024 eruption. These fissures passed under a protective barrier and opened very close to the town of Grindavík. The lava flows produced from these January 2024 fissures destroyed several properties in Grindavík.

### Discussion

**Fig. 8:**
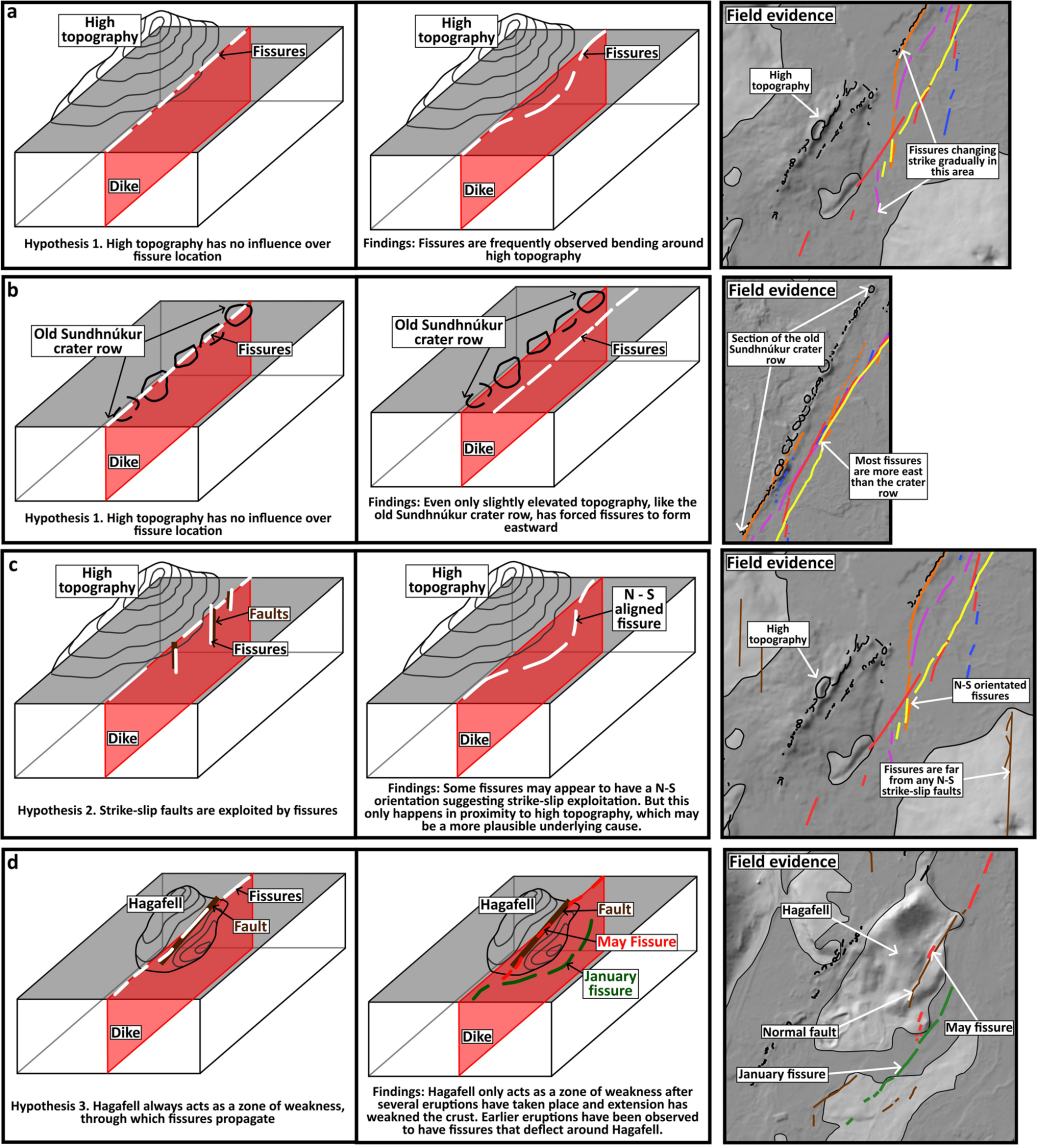
Conceptual schematics illustrating the hypotheses proposed by Jenness and Clifton (2009) for fissure location and propagation, compared against field evidence from the December 2023–September 2024 eruptions of the Svartsengi Volcanic System. In each row, the left panel shows the original hypothesis, the middle panel summarises the observed fissure behaviour, and the right panel presents representative field evidence from Fig. 7. (a) Hypothesis 1: high topography does not influence fissure location. Observations show fissures are frequently deflected and bend around areas of high topography, rather than propagating directly above the dike. Field evidence comes from Fig.7d. (b) Hypothesis 1 applied to subtle topography: even low-relief features such as the old Sundhnúkur crater row influence fissure placement. Observed fissures are displaced eastward relative to the crater row, indicating deflection by elevated topography. Field evidence comes from Fig.7c. (c) Hypothesis 2: strike-slip faults are exploited by fissures. Some fissures appear to have an N–S orientation suggestive of strike-slip control, but this alignment only occurs in proximity to high topography, indicating that topographic deflection is a more plausible underlying cause than fault exploitation. Field evidence comes from Fig.7d. (d) Hypothesis 3: Hagafell acts as a persistent zone of weakness. Observations show that Hagafell only behaves as a zone of weakness after repeated eruptions and progressive crustal extension, with January 2024 fissures deflecting around Hagafell and May 2024 fissures exploiting a normal fault on its flank. Field evidence comes from Fig.7e. Mapped fissures, faults, crater rows, and lava outlines in the field evidence panels follow the same colour scheme as Fig.7. White dashed lines indicate fissure traces, red volumes represent the inferred dike, grey shading indicates topography, and brown lines show mapped faults.

The 2350 B.P. Sundhnúkur crater row, mapped by Jenness and Clifton (2009) provides an opportunity to compare the December 2023 – August 2024 fissures with older crater rows and faults that have since been buried by lava flows. Jenness and Clifton (2009) proposed several controls on fissure location that can now be evaluated using the recent eruptions: (i) deflection by surface topography; (ii) exploitation of N – S strike-slip faults, and (iii) repeated fissure propagation through Hagafell as a zone of weakness (Fig. 8).

#### Deflection by crustal loading

Previous work has suggested that the gravitational load of volcanic edifices can modify the shallow crustal stress field and influence dike propagation (Klügel et al. 2005). Even stress changes of only a few bars can alter the direction of dike and fissure propagation (Walter and Amelung 2006). Building on this, Jenness and Clifton (2009) proposed that subtle topographic variations within the Svartsengi lava field could deflect fissures away from elevated topography.

In contrast, other studies have argued that in extensional settings, such as the Reykjanes Peninsula, magma may be preferentially drawn towards topographic highs rather than low-lying areas (Acocella et al. 2023). Similarly, Gaffney and Damjanac (2006) have suggested that where elevated topography is aligned parallel to a dike, fissures are more likely to open onto the ridge than into adjacent low terrain.

In this study, we observe that fissures are commonly deflected around topography, even where that topography is oriented parallel to the inferred dike. During the 2023–2024 eruptions, fissures were deflected eastward away from the old, dike-parallel Sundhnúkur crater row (Fig. 8a), and around Hagafell unless they exploited pre-existing normal faults (Fig. 8d). Between Sýlingarfell and Stóra Skógafell, fissures opened east of the Sundhnúkur crater row (Fig. 8b), whereas north of Stóra Skógafell the August 2024 fissure closely overlaps the crater row (Fig. 7a). This contrast may reflect differences in lava accumulation and subtle topography, with the larger volume of the 2–3 ka lava flows between Sýlingarfell and Stóra Skógafell producing a greater perturbation to the shallow stress field (Jenness and Clifton 2009; Fig. 8a, b). In some areas (Fig. 7c), fissures from successive eruptions are progressively displaced eastward, suggesting that lava emplacement and cone construction during earlier eruptions influenced the locations of later fissures. During the January 2024 eruption, fissure propagation was also locally inhibited by a 5–6-m-high protective barrier, forcing the fissure to re-open on the opposite side and form a new segment.

These observations are supported by the conceptual and experimental models of Hurley et al. (2025), who investigated dike and fracture propagation beneath Mt Þorbjörn during the 2023–2024 unrest. Their results show that strong topographic gradients modify the shallow stress field, causing ascending dikes to encounter variable confining stresses that can bend magma pathways and locally inhibit eruption. They further suggest that fractures on elevated terrain are less likely to become magma-filled because increased confining pressure promotes magma stalling. This provides a quantitative framework for the observed tendency of fissures to avoid high topography within the Svartsengi Volcanic System (Fig. 8a).

#### Exploitation of strike-slip faults

Strike-slip faults on the Reykjanes Peninsula are oriented approximately N–S (Sæmundsson et al. 2020), so fissures exploiting them should have a similar orientation. No fissure segment has opened directly along a mapped strike-slip fault during the 2023–2024 eruptions, although several segments, particularly south of Sýlingarfell, strike N-S (Fig. 8c). However, topographic deflection may provide an alternative explanation because fissure orientations progressively rotate from NNE–SSW to N–S close to elevated terrain (Fig. 8c). This suggests that topography, rather than fault exploitation, may control the observed alignment. In contrast, eruptive vents during the 2021–2023 Fagradalsfjall eruptions were largely emplaced along prominent N–S strike-slip faults (Hjartadóttir et al., 2023).

#### Hagafell zone of weakness

Jenness and Clifton (2009) proposed that Hagafell hosts a persistent zone of weakness, through which magma has repeatedly risen, as evidenced by Sundhnúkur craters that traverse Hagafell’s western edge along conjugate fractures. However, fissure segments during the January 2024 eruption were deflected around Hagafell, whereas a fissure segment in May 2024 opened on its eastern flank by exploiting a normal fault (Fig. 8d). These observations suggest that Hagafell may initially act as a topographic barrier to fissure propagation until sufficient extension or weakening has occurred later into the rifting episode (Fig. 8d).

#### Fissure orientations

Fissure orientations (Fig. 7f) vary both between and within eruptions. The December 2023 and January 2024 eruptions exhibit the greatest variability in fissure segment orientations and are also the most strongly segmented. This suggests that repeated extension and magma intrusion progressively localised strain within the fissure field, allowing later fissures to propagate along a more consistent structural trend. Between Sýlingarfell and Stóra Skógafell, where eruptive activity has been concentrated, fissures show the most consistent NE-SW orientation, suggesting the development of a defined zone of weakness. The fissure segments from the March 2024 eruption have a notably more eastward orientation compared to the other eruptions due to strong topographic influences close to the fissure, whereas the August 2024 fissure aligns most closely with the ~ 40° modal orientation.

The modal fissure bearing of the recent fissures is 5° farther south than that of the Sundhnúkur crater row (Fig. 7g). This difference is likely caused by topographic deflection of the new fissures away from the older crater row, suggesting that lava accumulation from previous eruptions can influence the location and orientation of subsequent fissures.

## Fire-fountains

### Results

**Fig. 9:**
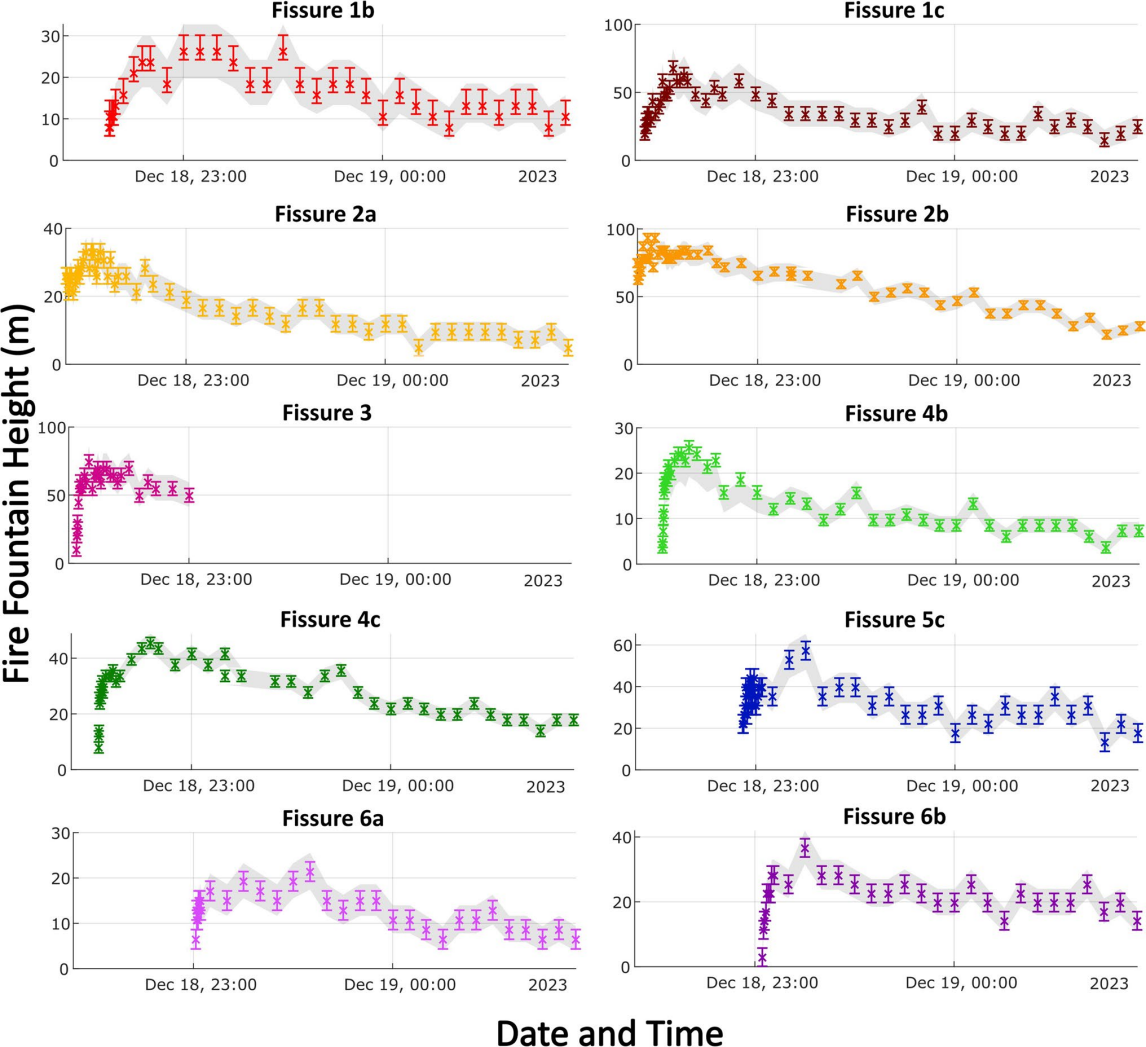
Fire-fountain height time series for individual fissure segments during the December 2023 eruption. Panels show measured fire-fountain heights for fissure segments 1b-c (reds), 2a-b (oranges), 3 (pink), 4b-c (greens), 5c (blue), and 6a-b (purples). Points indicate measured fire-fountain heights at each sampling interval, with vertical error bars representing measurement uncertainty derived from ruler precision and segment-specific scaling uncertainty. The grey shaded envelopes show the combined uncertainty from the ruler uncertainty and angle uncertainty associated with uncertain webcam locations. Time is shown as date and local time, with measurements beginning at the first observation of fountain activity for each segment. Differences in the start times and durations of individual time series reflect the progressive opening of fissure segments. Other fissure segments during the December eruption are excluded because their fire-fountains could not be measured from the webcam footage

**Fig. 10:**
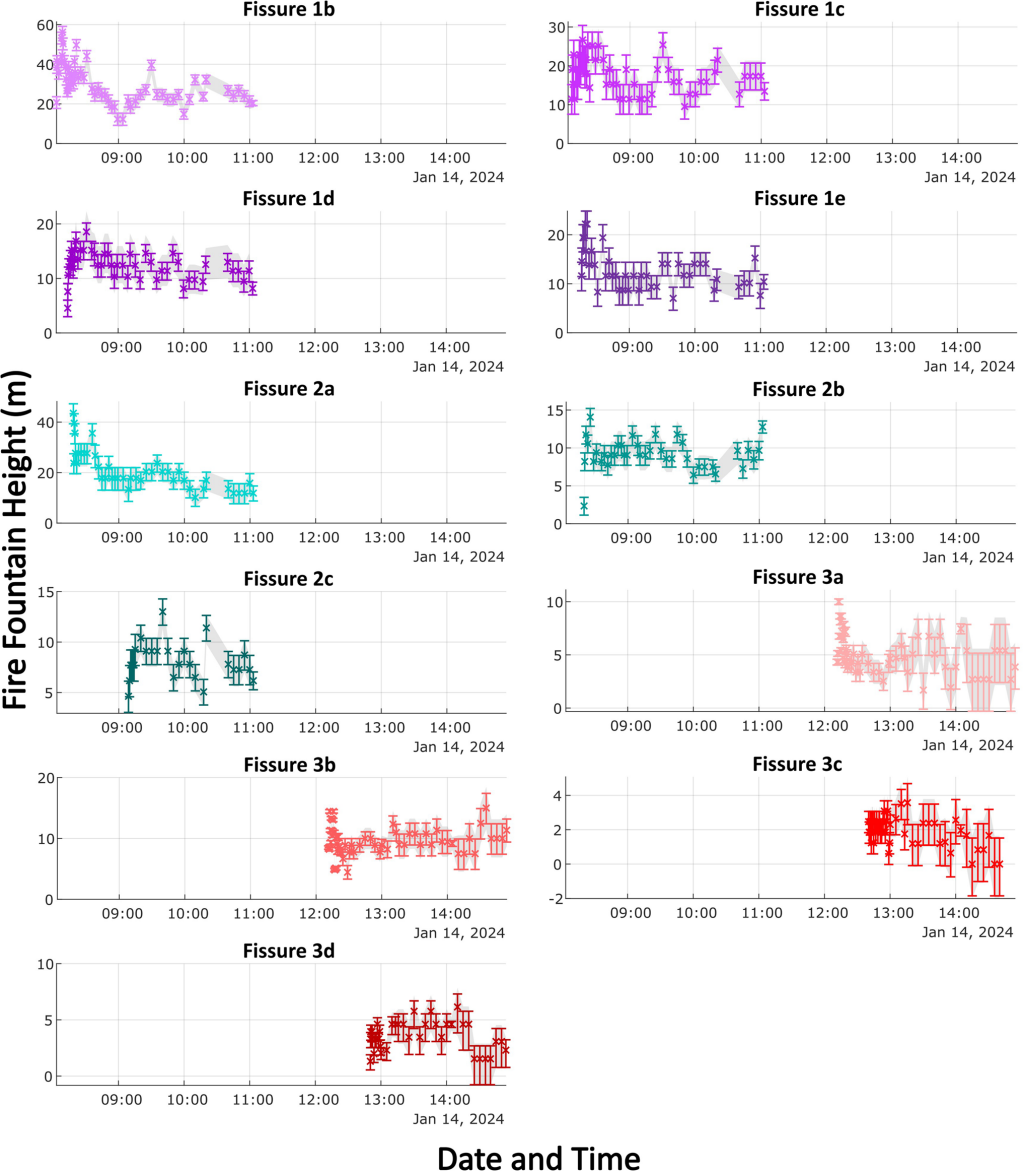
Fire-fountain height time series for individual fissure segments during the January 2024 eruption. Panels show measured fire-fountain heights for fissure segments 1b–1e (purples), 2a–2c (teals), and 3a–3d (reds). Points indicate measured fire-fountain heights at each sampling interval, with vertical error bars representing measurement uncertainty derived from ruler precision and segment-specific scaling uncertainty. The grey shaded envelopes show the combined uncertainty from the ruler uncertainty and angle uncertainty associated with uncertain webcam locations. Time is shown as date and local time, with measurements beginning at the first observation of fountain activity for each segment. Differences in the start times and durations of individual time series reflect the progressive opening of fissure segments. Other fissure segments during the January eruption are excluded because they could not be measured from the webcam footage

**Fig. 11:**
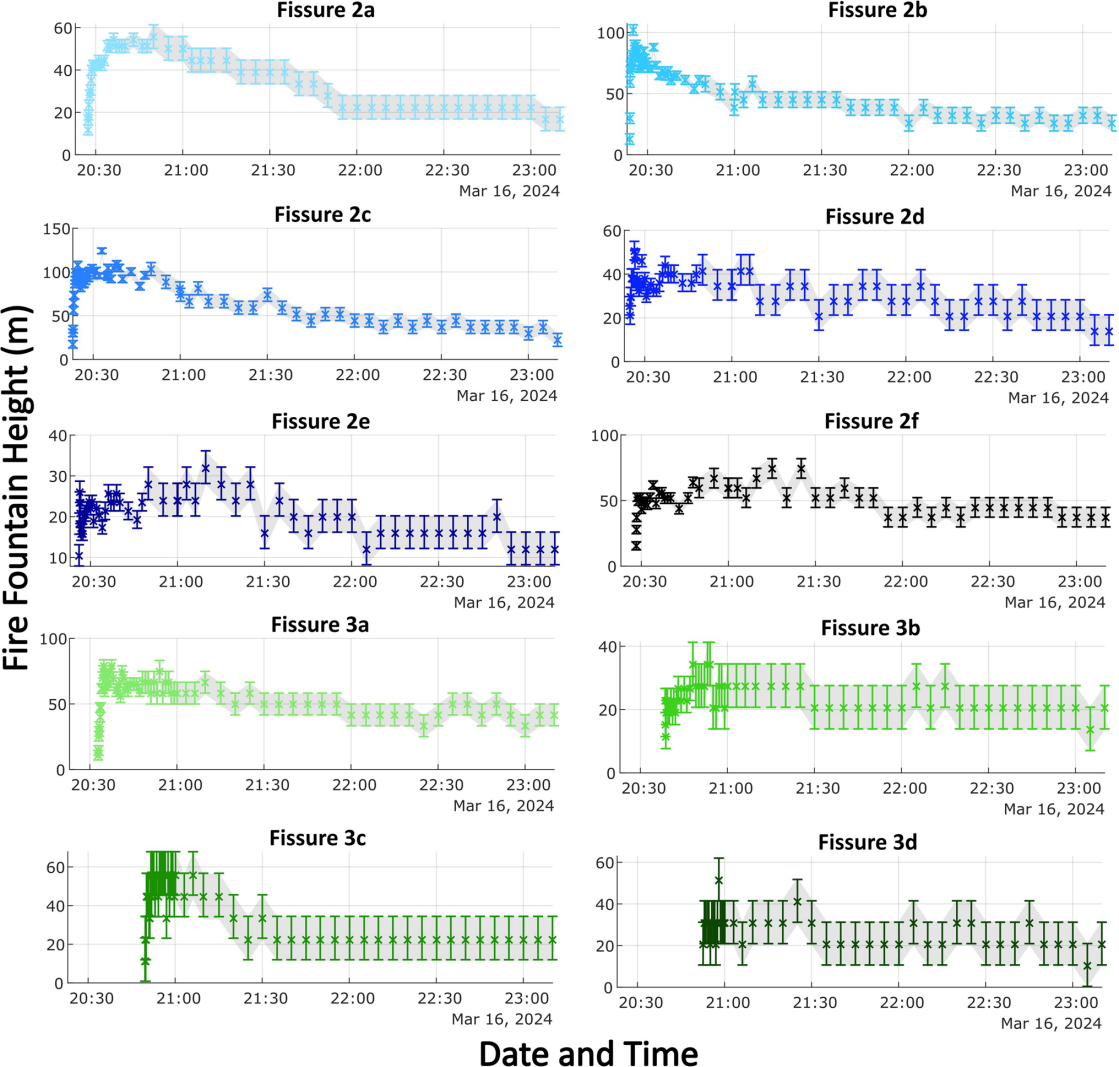
Fire-fountain height time series for individual fissure segments during the March 2024 eruption. Panels show measured fire-fountain heights for fissure segments 2a–2f (blues) and 3a–3d (greens). Points represent measured fire-fountain heights at each sampling interval, with vertical error bars indicating measurement uncertainty derived from ruler precision and scaling uncertainty. The grey shaded envelopes show the combined uncertainty from the ruler uncertainty and angle uncertainty associated with uncertain webcam locations. Time is shown as date and local time, with measurements beginning at the first observation of fountain activity for each segment. Differences in the start times and durations of individual time series reflect the progressive opening of fissure segments. Other fissure segments during the March eruption are excluded because they could not be measured from the webcam footage

**Fig. 12:**
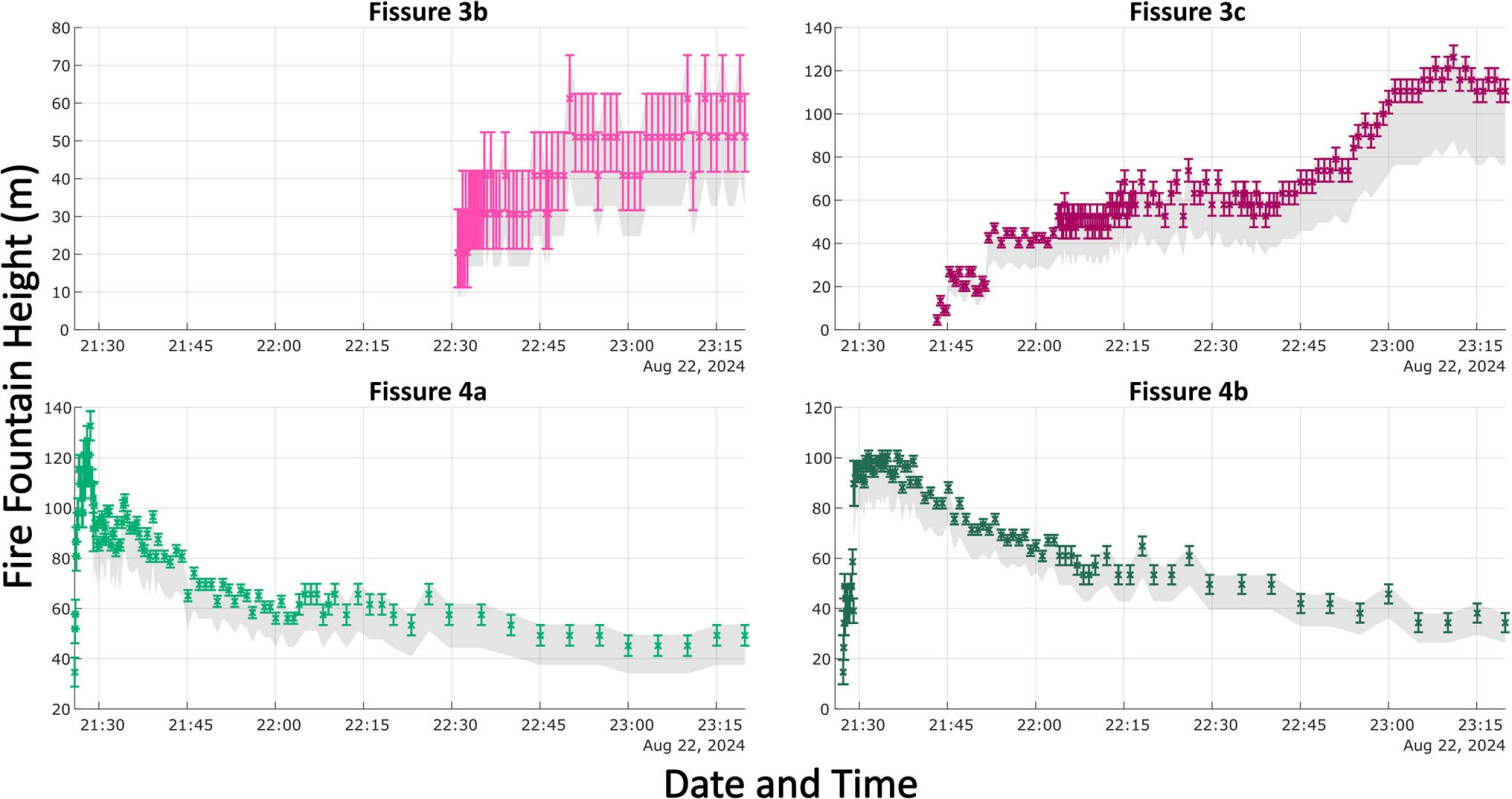
Fire-fountain height time series for individual fissure segments during the August 2024 eruption. Panels show measured fire-fountain heights for fissure segments 3b, 3c, 4a, and 4b. Points represent measured fire-fountain heights at each sampling interval, with vertical error bars indicating measurement uncertainty derived from ruler precision and scaling uncertainty. The grey shaded envelopes show the combined uncertainty from the ruler uncertainty and angle uncertainty associated with uncertain webcam locations. Angle uncertainty is highest in the August 2024 eruption because the webcam view was highly oblique to the fissures. Time is shown as date and local time, with measurements beginning at the first observation of fountain activity for each segment. Differences in the start times and durations of individual time series reflect the progressive opening of fissure segments. Other fissure segments during the August eruption are excluded because they could not be measured from the webcam footage

The early stages of each of the 2023–2024 Svartsengi Volcanic System eruptions were dominated by the ejection of a magmatic spray, forming fire-fountains. During each eruption, the fire-fountain heights varied between fissure segments (Figs. 9,10,11,12), so here we investigate the spatial and temporal height variation for segmented fissures.

#### Fire-fountain heights in the December 2023 eruption

Using footage from the December 2023 eruption, we measured fire-fountains from 10 of the 19 active fissure segments (Fig. 9). The camera view remained stable throughout the observation period, so measurement uncertainties were consistent. Measurements from fissure segment 3 were cut short because the oblique viewing angle meant that it was obscured from view as fissure segment 4a propagated.

Most fissure segments reached their maximum fire-fountain height within 15 min of propagation, although fire-fountains from fissure segments 6a and 1b peaked after 30 min. Following peak activity, fire-fountains generally declined over time. While most segments showed relatively uniform decay, fissure segments 1b, 5c, 6a, and 6b exhibited fluctuations in fire-fountain height.

The highest fire-fountains were consistently recorded from fissure segment 2b, including the eruption maximum of 93 m at 22:27, 18 December (local time). This was the first fissure segment to open during the eruption. The smallest fire-fountains were consistently observed at fissure segments 2a, 4b, and 6a.

#### Fire-fountain heights in the January 2024 eruption

Fire-fountain heights were measured for 11 of the 13 active fissure segments of the January 2024 eruption (Fig. 10). The camera footage was less stable than in December 2023, with frequent movement and zoom changes, requiring repeated recalibration of scaling factors. At around 10:30, 14 January, the cameras shifted to follow advancing lava flows, interrupting fire-fountain measurements for roughly 20 min.

Fissure propagation occurred in two stages. Phase 1 began near Hagafell at ~ 08:00, 14 January and involved fissures 1 and 2, while Phase 2 began near Grindavík at ~ 12:00, 14 January and involved fissure 3.

During Phase 1, the maximum recorded fire-fountain height was 56 m at 08:09, 14 January, on fissure segment 1b. This was the first fissure segment to open during the eruption. Fire-fountain heights fluctuated more than in the December 2023 eruption, and segments 1b, 1c, 2a and 2c showed a short-lived increase in intensity at ~ 09:30, 14 January. These variations obscure any clear decay trend. All segments except 2c reached their maximum height within 15 min of propagation. Segment 2c reached a peak after 30 min based on a single measurement that coincided with the same period of heightened activity observed in other segments.

During Phase 2, fissure segments were shorter and fire-fountains smaller than in Phase 1. The maximum height recorded was 15 m on fissure segment 3b at 14:36, 14 January, and the mean height over the 3-h observation period was 5.3 m. Interpretation of this phase was limited by the small fountain sizes and the zoomed-out camera view. Consequently, trends in Phase 2 remain uncertain.

#### Fire-fountain heights in the March 2024 eruption

Fire-fountains were measured on 10 out of the 14 active fissure segments during the March 2024 eruption (Fig. 11). The webcam footage of this eruption was of lower quality because it was zoomed out so that the whole fissure could be seen at once. The footage was also often unstable. This increased the uncertainties of the fire-fountain measurements, most notably on fissure segments 3b, 3c and 3d, where the uncertainties were around 20 m, limiting the ability to interpret temporal variation.

The maximum fire-fountain height recorded was 124 m from fissure segment 2c at 20:33, 16 March. This fissure segment consistently produced the tallest fountains during the eruption. This was the first fissure segment to open during the eruption. Fire-fountain heights generally decreased more slowly in the March 2024 eruption compared to the December 2023 eruption. Many fissure segments had highly variable fire-fountain heights that did not decrease significantly overall. Fissure segments 2a, 2b, 2c, and 3c displayed a clear, uniform rise and decay pattern, reaching their maximum heights within 10 to 15 min of fissure segment propagation before decaying.

#### Fire-fountain heights in the August 2024 eruption

During the August 2024 eruptions, it was only possible to measure fire-fountains on 4 of the 14 active fissure segments because the camera was positioned too far south so could not capture fissure segments 1 and 2 and topographic highs obscured fissure 5 from view (Fig. 12). The camera from which the measurements were taken was very oblique to the fissure; this means that there was some visible overlap between the ends of fissure segments, and angular uncertainty had a larger impact on these results. The only camera with a less oblique view had the fire-fountains obscured by fumes. The footage was generally well zoomed and clear, but the measurement window was cut short because the camera operator moved the view to follow the lava flows, meaning the fire-fountains could no longer be seen.

Fire-fountain heights during the August 2024 eruption were consistently higher than in the other measured events. Fissure segments 4a and 4b show a clear rise and decay pattern, reaching their maximum heights within approximately 10 min of fissure segment propagation. The tallest fire-fountain during the eruption was measured at 133 m on fissure segment 4a at 21:29, 22 August. This was the first fissure segment to open during the eruption. Fissure segment 3b exhibited a more gradual increase in fire-fountain height, with heights eventually plateauing at 50 m. However, there are considerable uncertainties associated with 3b because its fire-fountains are so small on screen, and the tail end of the fissure is obscured by 3c. Fissure segment 3c shows a different temporal evolution in fire-fountain height than the other measured fissure segments. It has a slow increase in height immediately after propagation, but fire-fountain heights begin to increase rapidly at 22:40, 22 August, reaching a peak height of 126 m at 23:11, 22 August, before declining.

#### Fitting a rise-decay model

**Fig. 13:**
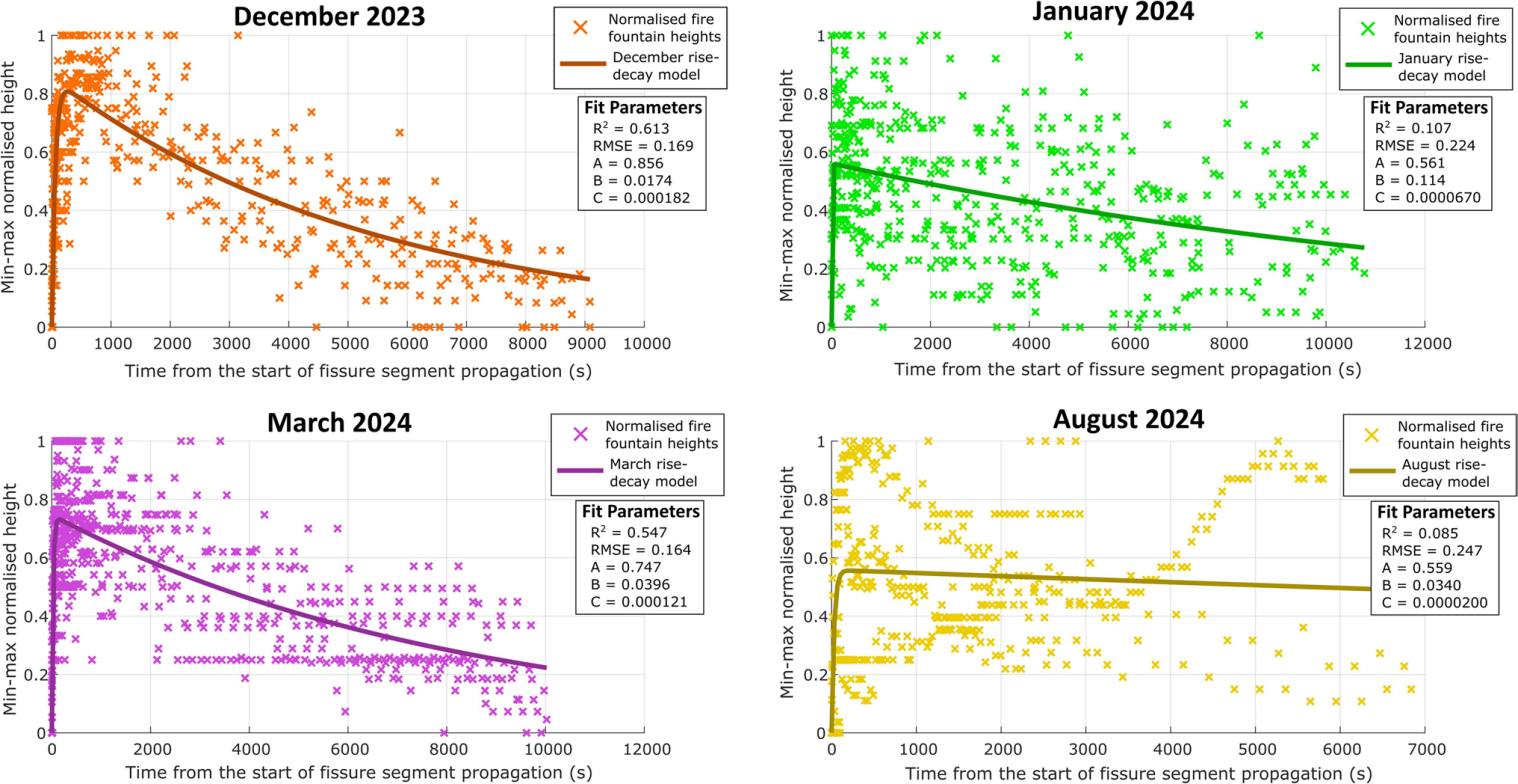
Normalised fire-fountain height measurements and rise–decay model fits for individual eruptions. Panels show normalised fire-fountain heights plotted against time, with points representing measured values. Coloured solid lines show the best-fitting rise–decay model for each eruption. Reported ${\mathrm{R}}^{2}$ and RMSE values indicate the goodness of fit for the eruption-specific model and for the global model evaluated against each eruption’s data. The A, B, and C coefficients for the rise-decay model are also shown. More details shown in table.S2

We fitted rise-decay models (Eq. (2)) to fire-fountain height measurements from each eruption to describe the initial increase and subsequent decline in fountain height:

2 $$h\left(t\right)=A\cdot\left(1-e^{-Bt}\right)\cdot e^{-Ct}$$

where A is the curve amplitude, B is the rise rate following fissure opening, and C is the decay rate after the peak (Fig. 13). The models are intended as descriptive summaries of typical fire-fountain behaviour.

For each fissure segment, the time of first observed opening was defined as t=0. Fire-fountain heights were normalised to the range [0, 1], and the normalised time series from all segments within an eruption were pooled. A single rise–decay model was then fitted to each eruption, capturing eruption-scale trends while allowing individual segments to differ in absolute height.

Model coefficients (A, B, and C) were estimated by minimising the sum of squared residuals using MATLAB’s nonlinear regression function (fitnlm). A *t*-test was used to assess whether each coefficient differed significantly from zero. Model performance was evaluated using the coefficient of determination (*R*^2^; Eq. (3)) and root mean square error (RMSE; Eq. (4)).

3 $${R}^{2}=1-\frac{{SS}_{res}}{{SS}_{tot}}$$

4 $$RMSE= \sqrt{\frac{1}{n}\sum {({h}_{measured}-{h}_{fit})}^{2}}$$

where SS_res_ is the sum of squared residuals, and SS_tot_ is the total variance in the data, n is the number of fire-fountain measurements, h_measured_ are the observed normalised heights, and h_fit_ are the modelled values. Higher *R*^2^ values and lower RMSE values indicate better model fit (Table S2).

Fire-fountain measurements from the same fissure segment are temporally correlated, and segments within an eruption share common magma supply and stress conditions. Consequently, the pooled rise–decay fits are not statistically independent. The fitted models are therefore interpreted as descriptions of eruption-scale fire-fountain evolution, rather than as the basis for formal hypothesis testing.

Fire-fountains measured during the December 2023 and March 2024 eruptions are well described by rise-decay models, whereas those from the January 2024 and August 2024 eruptions show substantially poorer fits. The August 2024 rise-decay model is not statistically significant (Table S2). Despite these differences, the tallest fire-fountain in each eruption consistently originated from the first fissure segment to open.

Although the August 2024 fire-fountain heights do not follow a statistically significant rise-decay model overall, fire-fountains on fissure segments 4a and 4b individually exhibit clear rise-decay behaviour. This suggests that when magma supply remains relatively stable, and dike propagation is complete before the onset of an eruption, fire-fountains evolve according to a predictable rise-decay pattern. Departures from this pattern during the January 2024 and August 2024 eruptions may instead reflect continued dike propagation and variations in magma supply during the eruption.

### Discussion

#### Controls on fire-fountain height

Factors previously proposed to control fire-fountain height include magma volatile content, mass eruption rate, magma viscosity, vent size and source overpressure (Wilson and Head 1981). The influence of varying magma volatile content and viscosity between individual fissure segments cannot be evaluated here because suitable samples are not available. Consequently, this study focuses on physical rather than geochemical factors affecting fire-fountain heights.

Within a single eruption, geochemical parameters are unlikely to vary substantially between fissure segments since they are fed by the same dike. Assuming that these geochemical differences are relatively minor allows investigation of the effect of physical variations on the fire-fountain system, including fissure segment length, magma pressurisation, and temporal and spatial proximity to the eruption onset.

#### Fissure segment length

There is a significant positive correlation between fissure segment length and fire-fountain height during the January 2024 and March 2024 eruptions, with longer fissure segments generally producing taller fire-fountains (Table S1). This relationship is consistent with previous studies linking vent dimensions to eruption intensity. Wilson et al. (1980) demonstrated that eruption column height increases with vent size regardless of gas content, whereas changes in gas content at a constant vent size produce comparatively small variations in column height. Suzuki et al. (2020) showed that, under flow conditions typical of narrow fissures, plume height increases with fissure aspect ratio. Georgeais et al. (2024) found that mass eruption rate is proportional to gas-jet velocity, column density, and vent area, and argue that some plume heights observed at Stromboli require increases in vent area rather than changes in velocity or density alone. Together, these studies indicate that vent geometry exerts a strong control on plume height as larger openings can sustain greater mass eruption rates. Our findings, therefore, provide independent observational support for this interpretation.

In contrast, the December 2023 eruption shows a negligible correlation between fissure segment length and fire-fountain height. This may reflect the influence of the eruption onset, as the first fissure segment was relatively short but nevertheless produced the tallest fountain of the eruption. The prominence of this segment reduces the overall strength of the correlation.

Similarly, no significant correlation is observed during the August 2024 eruption due to localised increases in fire-fountain height on one fissure segment.

#### August 2024 increased fire-fountain height

Approximately one hour after eruption onset, fire-fountain heights on fissure segment 3c began to increase, while heights on the other fissure segments either plateaued or declined (Fig. 12). This increase on the penultimate northern fissure segment coincided with continued northward dike propagation and the opening of additional fissure segments, although propagation of these fissures was obscured in the webcam footage. Because the increase was restricted to a single fissure segment rather than occurring across all active segments, the process responsible was likely acting only locally.

Two mechanisms could explain the increased fire-fountain heights on fissure segment 3c: fissure widening and dike overpressure. Widening of the fissure segment, potentially through vent erosion, would increase the size of the surface vent and therefore the mass eruption rate, allowing a greater mass flux and taller fire-fountains (Burgisser and Degruyter 2015; Sahagian 2005; Piombo et al. 2016; Mitchell 2005).

Alternatively, overpressure within the dike would also increase the mass eruption rate and consequently fire-fountain heights (Wilson and Head 1981). The timing of the height increase on fissure segment 3c, coincident with continued northeastward dike propagation (Li et al. 2025; Parks et al. 2025), suggests that pressurisation near the propagating dike tip may have contributed to the observed increase. A similar process may explain the simultaneous increase in fire-fountain heights at 09:30 across multiple fissure segments during the January 2024 eruption as the dike continued propagating southwest (Fig. 10; Li et al. 2025; Parks et al. 2025). Pressure build-up at the distal ends of propagating dikes has been inferred from seismic and geodetic observations (e.g., Sigmundsson et al. 2015; Isken et al. 2025), and it is plausible that similar pressurisation during the January 2024 and August 2024 eruptions may have increased eruptive vigour and produced higher fire-fountains.

#### Spatial and temporal proximity to the start of the eruption

In all four of the measured eruptions, the highest fire-fountain measured during the eruption originated from the fissure segment that was first to open. Pressure in a magma reservoir is expected to drop during dike and fissure propagation as an eruption progresses (Einarsson 1991), a likely explanation as to why the paroxysm of the eruptions was reached on the first fissure segment to open, within the first minutes of the eruptions. Furthermore, the smallest fire-fountains observed in all the eruptions originated from the Phase 2 fissure segments in the January 2024 eruptions. These fissure segments opened more than 4 h after the start of the eruption and were separated from the rest of the January 2024 fissure segments by at least 500 m, spatially and temporally far from the start of the eruption.

#### Implications of the webcam network

The methods used in this study differ from those employed in many previous fire-fountain studies that relied on dedicated scientific webcam networks. In research-focused monitoring systems, camera placement, operation, and zoom are typically selected to optimise measurement quality. Cameras are commonly positioned approximately perpendicular to the expected fissure orientation to minimise perspective distortion and reduce the apparent overlap of fissure segments during propagation (e.g. Witt et al. 2018). Fixed camera locations and stable fields of view also allow observations to be calibrated consistently through time using a single calibration frame (e.g. Pasqualon et al. 2025).

These conditions enable automated image-processing techniques to measure fire-fountain heights continuously throughout an eruption. Previous studies have commonly used Sobel edge-detection methods on webcam footage that has been post-processed to enhance image contrast (Witt and Walter 2017; Jin-Yu et al. 2009). Automated approaches increase temporal sampling frequency and may reduce some uncertainties associated with manual measurements.

In contrast, the recreational webcam footage used here presents several limitations for quantitative analysis. The field of view and zoom frequently changed during eruptions, and many fissures were viewed from highly oblique angles. These factors complicated fire-fountain scaling and prevented the use of automated edge-detection methods. Consequently, the dataset is more temporally sparse than those derived from dedicated scientific monitoring systems. The measurements presented here should therefore be regarded as an opportunistic use of available footage rather than a comprehensive record of eruptive behaviour.

Several modifications to the webcam network around the Svartsengi Volcanic System could improve its suitability for future scientific studies while retaining its value for public viewing. Positioning additional cameras further north within the fissure field, particularly on Sýlingarfell, would provide viewing angles closer to perpendicular to the most common areas of fissure propagation as identified in this study. This location would offer sufficient elevation and a wide enough field of view to capture more of the fissure length while reducing the need for frequent camera movement. Maintaining at least one camera with a fixed field of view and stable zoom throughout eruptions would also facilitate automated image analysis. Such observations could provide higher temporal resolution measurements and allow the patterns identified here to be tested using more continuous datasets.

**Fig. 14:**
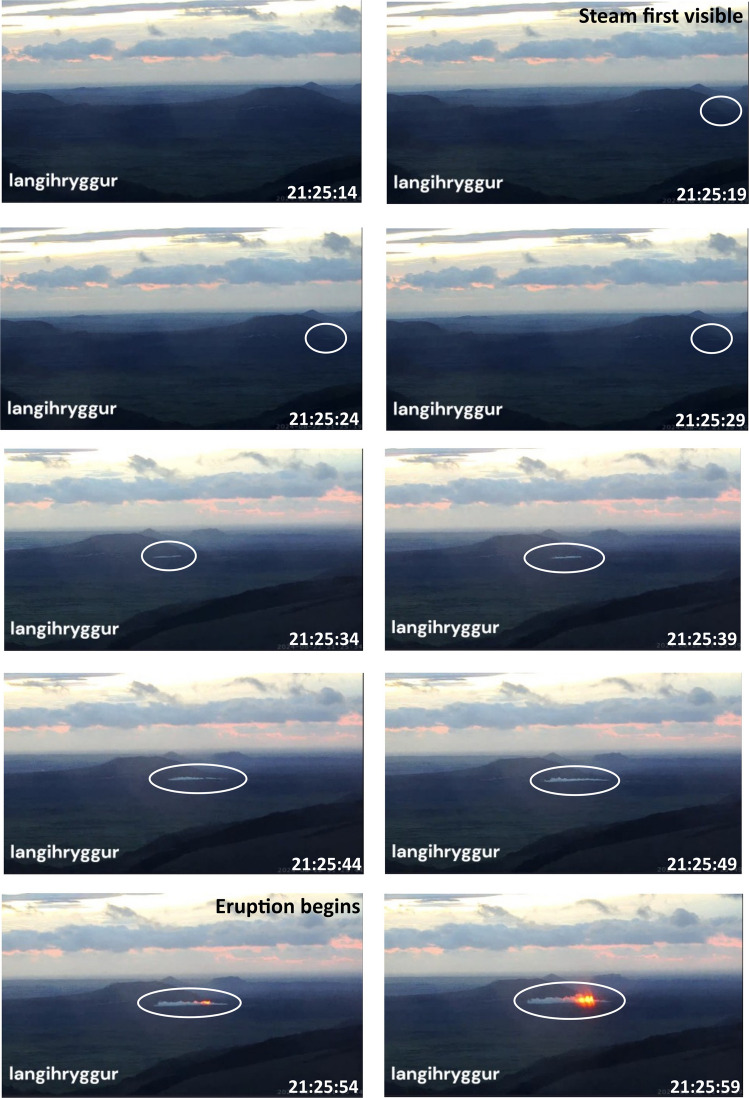
Time series of webcam images showing the appearance of steam before fissure opening and the onset of eruption. Images are from the Langihryggur webcam and are shown at ~ 5 s intervals. White ellipses highlight the location where steam first becomes visible, before transitioning to incandescent lava fountains. Steam emissions are first visible at 21:25:19, intensifying progressively, and are followed by the appearance of lava at 21:25:54, marking the onset of eruptive activity. Timestamps shown in the lower right of each panel indicate local time

Despite these limitations, the webcam network provided opportunities to observe additional aspects of eruptive behaviour. Because the cameras operate continuously, they captured conditions immediately preceding eruption onset. Footage from every eruption of the Svartsengi Volcanic System showed steam or fume emissions from the nascent fissure location before fissure opening, sometimes several minutes before eruption onset. During the August 2024 eruption, for example, steam was visible approximately 40 s before fissure opening (Fig. 14). The length of time for which these emissions could be observed depended on lighting conditions. Similar behaviour was reported during the Fagradalsfjall eruptions, where steam was visible for 15–90 s at night and 9–23 min in daylight before eruption onset (Hjartardóttir et al. 2023). These observations suggest that continuous webcam monitoring may help identify the location of impending fissure opening and, under favourable viewing conditions, provide a short-term indication of eruption onset.

## Conclusions

Fissure locations have previously been proposed to be influenced by pre-existing crustal weaknesses, crustal loading and topography. Our observations show that the relative influence of these factors varies across the Svartsengi fissure field (Fig. 2). In the south, Hagafell’s topography and structural weaknesses both deflect and preferentially facilitate fissure propagation. In the centre of the lava field, fissures have formed progressively east of the Sundhnúkur crater row. Regional topography causes lava to flow preferentially westward, resulting in increasing lava loading to the west and progressively eastward fissure propagation during successive eruptions. In the north, the absence of significant topographic barriers allows fissures to align perpendicular to the least compressive stress and propagate along normal faults and the Sundhnúkur crater row, enabling re-exploitation of this structural weakness. Where present, topography and faults exert the strongest controls on fissure location. Understanding these influences improves fissure location forecasts and informs hazard mitigation.

Fire-fountain heights are primarily controlled by fissure segment length, dike propagation, and proximity to eruption onset. Longer fissure segments generally produce higher fire-fountains, except where the first segment to open is small. In every eruption, the first segment to open produced the highest fire-fountains. Variations during the January 2024 and August 2024 eruptions highlight the influence of dike pressurisation on mass eruption rate. In January 2024, several fissure segments experienced concurrent increases in fire-fountain height, and in August 2024, one segment showed progressively increasing heights coincident with continued northward dike propagation. Differences in magma storage conditions and volatile content may also contribute to variations in fire-fountain height between eruptions and could be assessed through future analysis of lava samples.

Strengthening the webcam network would improve future monitoring of Svartsengi Volcanic System eruptions. Additional cameras should be installed further north, and at least some cameras should maintain fixed viewing geometries throughout eruptions. Continuous, high-resolution monitoring would improve the characterisation of eruptive processes and support the development of forecasting models. With eruptions on the Reykjanes Peninsula expected to continue for decades to centuries (Sæmundsson et al. 2020), sustained monitoring remains necessary to improve eruption forecasting and hazard management.

## Supplementary Information

Below is the link to the electronic supplementary material.

Supplementary file1 (DOCX 8.53 MB)

Supplementary file2 (XLSX 3586 KB)

## Data Availability

Data is available to view in the data collection spreadsheet supplementary file. This includes a log of the webcam data, notes on camera quality, notes on fissure propagation which were used to identify fissure segments, fire fountain measurements including original on screen measurements and scaled heights, ruler uncertainty and angle uncertainty quantification, parameters used to assess correlations, comparison with automated measurement methods, and a collection of fire fountain heights that have been remeasured in Inkscape for reference.
