# Supplementary material for: Fissure locations and fire-fountain dynamics during the December 2023–September 2024 Svartsengi Volcanic System eruptions, Iceland, from aerial imagery and recreational webcam footage
Source: Bull Volcanol. 2026 Jul 11;88(8):89. doi: 10.1007/s00445-026-02006-3 (PMC13356074; doi:10.1007/s00445-026-02006-3)
Supplement: Supplementary file 1 — Supplementary file1 (DOCX 8.53 MB) [file 445_2026_2006_MOESM1_ESM.docx]

Supplementary materials

[Supplementary file 2: data collection spreadsheet 1](#_Toc233810725)

[Supplementary Video: August 2024 Svartsengi Volcanic System Eruption Fire Fountains 1](#_Toc233810726)

[Table.S1: Fire-fountain height correlations 1](#_Toc233810727)

[Table.S2: Fit of the rise-decay models 2](#_Toc233810728)

[Table.S3: Eruption duration, erupted volume, areas of lava flows and total fissure length 3](#_Toc233810729)

[Table.S4: Additional statistical tests on potential controlling factors of eruption characteristics 3](#_Toc233810730)

[Table.S5: Log of webcam footage 4](#_Toc233810731)

[Table.S6: Fissure segment opening times 5](#_Toc233810732)

[Fig.S1: Fissure segment labels 9](#_Toc233810733)

[Fig.S2: Ruler uncertainty explanation 16](#_Toc233810734)

[Fig.S3: Angular uncertainty explanation 17](#_Toc233810735)

[Figure.S4: Measurement and scaling protocol for fire-fountains 18](#_Toc233810736)

Supplementary file 2: data collection spreadsheet

Includes:

- Log of webcam data
- Camera quality notes
- Fissure propagation notes, timing and speeds. (assisted in identifying fissure segments)
- Fire-fountain measurements: on screen measurements, scaling factors for each fissure segment through time, scaled up fire-fountains
- Ruler uncertainty quantification
- Angle uncertainty quantification
- Parameters used to assess correlations

Supplementary Video: August 2024 Svartsengi Volcanic System Eruption Fire Fountains

This video is at ×16 playback speed. It includes webcam footage from MBL Webcam1 and LFI Langihryggur to demonstrate the 2 main viewing angles of most of the webcams: on Mount Þorbjorn, looking northeast, and on the east side of the lava field, looking west. Fire-fountain heights are plotted alongside the video.

Access via: <https://doi.org/10.5281/zenodo.20759139>

Table.S1: Fire-fountain height correlations

| ***Maximum fire-fountain height vs fissure segment length*** | **Eruption** | **Pearson’s R value** | **Correlation strength** | **Significant?** |
| --- | --- | --- | --- | --- |
|  | *December 2023* | 0.2965 | Negligible | No |
|  | *January 2024* | 0.6604 | Moderate | Yes |
|  | *March 2024* | 0.8730 | Strong | Yes |
|  | *Total mean (excluding August 2024)* | 0.8557 | Strong | Yes |
|  | *August 2024* | 0.2570 | Negligible | No |
| ***Distance between the first fissure segment to open, and the fissure segment from which the fire-fountain is measured vs maximum fire-fountain height*** | *December 2023* | 0.5134 | Moderate | No |
|  | *January 2024* | 0.7624 | Strong | Yes |
|  | *March 2024* | 0.4073 | Weak | No |
| ***Time from start of eruption vs maximum fire-fountain height*** | *December 2023* | 0.4067 | Weak | No |
|  | *January 2024* | 0.7206 | Strong | Yes |
|  | *March 2024* | 0.3645 | Weak | No |
| ***Table.S1: Correlation between fire-fountain height and various physical controlling factors.***  *Pearson’s R value of the correlation between maximum fire-fountain heights and fissure length, distance from starting fissure and time from the start of eruption. \|R\| < 0.3 negligible; \|R\| < 0.5 weak; \|R\| < 0.7 moderate; \|R\| < 0.9 strong; \|R\| > 0.9 very strong. Significant if p < 0.05* | | | | |

Table.S2: Fit of the rise-decay models

|  | **All fire-fountains** | **December** | **January** | **March** | **August** |
| --- | --- | --- | --- | --- | --- |
| **A** | 0.679 | 0.856 | 0.561 | 0.747 | 0.559 |
| **B** | 0.0374 | 0.0174 | 0.114 | 0.0396 | 0.0340 |
| **C** | 0.000108 | 0.000182 | 0.0000670 | 0.000121 | 0.0000200 |
| **Is the rise-decay model significant?** | Yes | Yes | Yes | Yes | No |
| **R^2^ value for the rise-decay model fit** | 0.304 | 0.613 | 0.107 | 0.547 | 0.085 |
| **RMSE value for the rise-decay model fit** | 0.211 | 0.169 | 0.224 | 0.164 | 0.247 |
| ***Table.S2: Rise decay model coefficients and statistics***  *A, B, C: the value of the constants in the rise decay model for each eruption and all the eruptions combined. The results from the T-test on the significance of the coefficients of the rise-decay model are shown.*  *R^2^ and RMSE values for each rise-decay model*  *The colour indicates whether this is a good fit (green) or a weak fit (red).* | | | | | |

Table.S3: Eruption duration, erupted volume, areas of lava flows and total fissure length

| Eruption | Dates | Volume erupted | Area of lava flows | Total fissure length |
| --- | --- | --- | --- | --- |
| December 2023 | 18 December – 21 December 2023 | 12 million m^3^ | 3.4 km^2^ | 4 km |
| January 2024 | 14 January – 16 January 2024 | 2 million m^3^ | 0.7 km^2^ | 0.9 and 0.1 km |
| February 2024 | 8 February – 9 February 2024 | 13 million m^3^ | 4.0 km^2^ | 3 km |
| March 2024 | 16 March – 8 May 2024 | 35 million m^3^ | 6.2 km^2^ | 2.9 km |
| May 2024 | 29 May – 22 June 2024 | 45 million m^3^ | 9.3 km^2^ | 2.4 km |
| August 2024 | 22 August – 5 September 2024 | 61 million m^3^ | 15.8 km^2^ | 3.9 km |
| *Table.S3:* *Dates, erupted volume, lava flow area and total fissure length for the 6 eruptions studied in this paper. Júlíusdóttir (2024)* | | | | |

Table.S4: Additional statistical tests on potential controlling factors of eruption characteristics

| 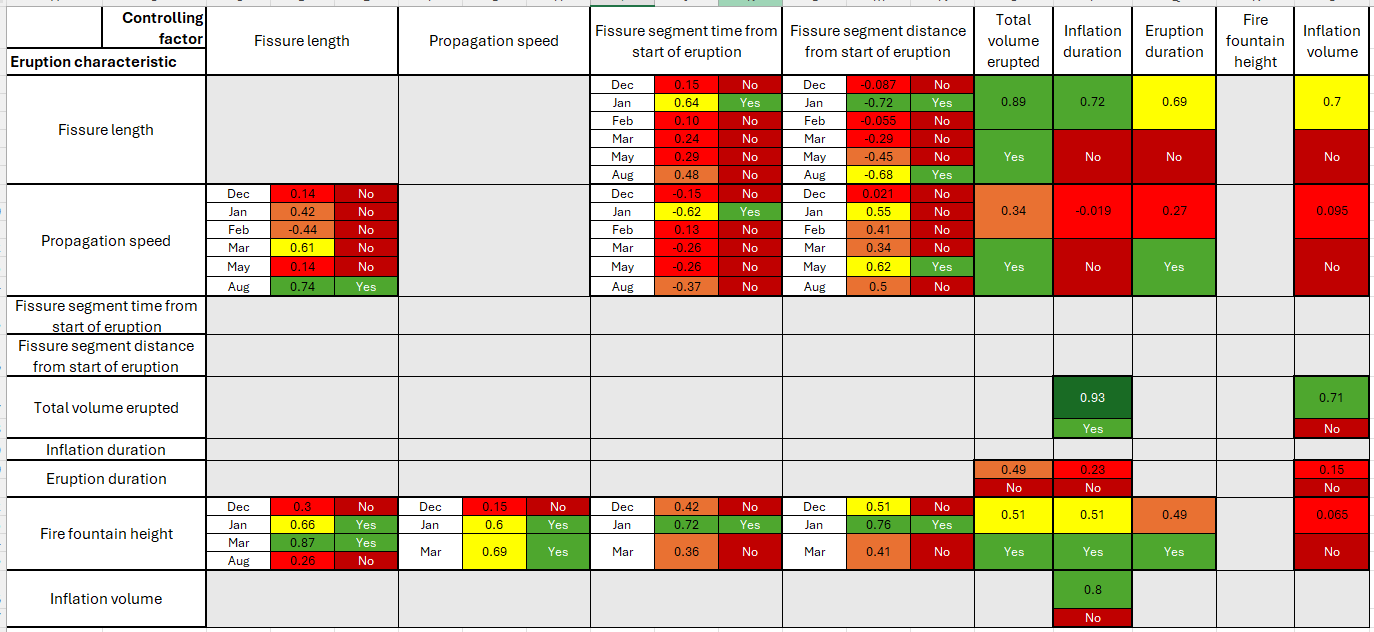 |
| --- |
| Colour explanations:  Significance of correlation Pearson’s R value  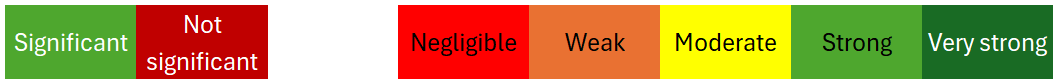 |
| ***Table.S4:*** *Additional statistical tests on potential controlling factors of eruption characteristics: fissure length, propagation speed, fissure segment opening time after start of eruption, fissure segment distance from the starting location of the eruption, total erupted volume, inflation duration, eruption duration, inflation volume*. |

Table.S5: Log of webcam footage

| **Eruption** | **Webcam** | **Resolution** | **Frame rate (FPS)** | **Start date** | **Start time** | **End date** | **End time** | **Used?** |
| --- | --- | --- | --- | --- | --- | --- | --- | --- |
| ***December 2023***  *18/12/23 – 21/12/23* | LFI langihryggur | 1080p | 25.00 | 18/12/2023 | 23:00 | 21/12/2023 | 00:00 | **Yes** |
|  | MBL webcam 1 | 1080p | 24.99 | 18/12/2023 | 22:00 | 21/12/2023 | 00:00 | **Yes** |
|  | MBL webcam2x | 1080p | 24.94 | 18/12/2023 | 22:00 | 21/12/2023 | 00:00 | Not as good as others |
| ***January 2024***  *14/01/24 – 16/01/24* | LFI reyjanes2 | 1080p | 25.00 | 14/01/2024 | 10:00 | 16/01/2024 | 06:00 | **Yes** |
|  | LFI visir 2 | 1080p | 25.00 | 14/01/2024 | 08:00 | 16/01/2024 | 01:00 | Poor quality |
|  | MBL webcam1 | 1080p | 25.00 | 14/01/2024 | 07:00 | 16/01/2024 | 11:00 | **Yes** |
|  | MBL webcam2x | 1080p | 25.00 | 14/01/2024 | 07:00 | 16/01/2024 | 11:00 | **Yes** |
|  | RUV4 | 1080p | 24.94 | 14/01/2024 | 06:00 | 14/01/2024 | 19:00 | **Yes** |
| ***February 2024***  *08/02/24 – 08/02/24* | LFI langihryggur | 1080p | 25.00 | 08/02/2024 | 06:00 | 09/02/2024 | 09:00 | **Yes** |
|  | MBL webcam1 | 1080p | 25.00 | 08/02/2024 | 06:00 | 09/02/2024 | 09:00 | **Yes** |
|  | MBL webcam2x | 1080p | 25.00 | 08/02/2024 | 06:00 | 09/02/2024 | 09:00 | **Yes** |
|  | RUV4 | 1080p | 25.00 | 08/02/2024 | 06:00 | 09/02/2024 | 02:00 | **Yes** |
|  | RUV5 | 1080p | 25.00 | 08/02/2024 | 06:00 | 09/02/2024 | 02:00 | Not needed |
| ***March 2024***  *16/03/24 – 09/05/24* | LFI grindavik | 1080p | 25.00 | 16/03/2024 | 20:00 | 16/03/2024 | 23:00 | Facing wrong direction |
|  | LFI langihryggur | 1080p | 25.00 | 16/03/2024 | 20:00 | 07/04/2024 | 00:00 | **Yes** |
|  | LFI reykjanes2 | 1080p | 25.00 | 16/03/2024 | 19:00 | 17/03/2024 | 13:00 | Too zoomed in |
|  | LFI reykjanes3 | 1080p | 25.00 | 16/03/2024 | 19:00 | 16/03/2024 | 22:00 | Same LOS as Langihryggur |
|  | LFI svartsengi | 1080p | 21.29 | 16/03/2024 | 20:00 | 16/03/2024 | 02:00 | Facing wrong direction |
|  | LFI thorbjorn2 | 1080p | 25.00 | 16/03/2024 | 20:00 | 24/04/2024 | 00:00 | **Yes** |
|  | LFI visir | 1080p | 25.00 | 16/03/2024 | 20:00 | 16/03/2024 | 22:00 | Facing wrong direction |
|  | LFI visir2 | 1080p | 25.00 | 16/03/2024 | 20:00 | 16/03/2024 | 22:00 | Facing wrong direction |
|  | MBL webcam1 | 1080p | 25.00 | 16/03/2024 | 20:00 | 16/03/2024 | 09:00 | Same as Thorbjorn |
|  | MBL webcam2 | 1080p | 25.00 | 16/03/2024 | 19:00 | 19/03/2024 | 22:00 | Cannot see propagation |
|  | MBL webcam2x | 1080p | 25.00 | 16/03/2024 | 19:00 | 16/03/2024 | 10:00 | Too zoomed out |
|  | MBL webcam3 | 1080p | 25.00 | 16/03/2024 | 19:00 | 16/03/2024 | 23:00 | Facing wrong direction |
|  | MBL webcam11 | 1080p | 25.00 | 16/03/2024 | 19:00 | 16/03/2024 | 22:00 | Poor quality |
| ***May 2024***  *29/05/24 – 22/06/24* | LFI reykjanes2 | 1080p | 25.00 | 29/05/2024 | 12:00 | 31/05/2024 | 10:00 | **Yes** |
|  | LFI thorbjorn2 | 1080p | 25.00 | 29/05/2024 | 12:00 | 31/05/2024 | 13:00 | **Yes** |
|  | MBL webcam2 | 1080p | 25.00 | 29/05/2024 | 12:00 | 05/06/2024 | 00:00 | **Yes** |
|  | MBL webcam2x | 1080p | 25.00 | 29/05/2024 | 17:00 | 05/06/2024 | 00:00 | Facing wrong way |
|  | MBL webcamA | 1080p | 25.00 | 29/05/2024 | 12:00 | 04/06/2024 | 04:00 | Yes |
| ***August 2024***  *22/08/24 – 06/09/24* | LFI langihryggur | 1080p | 25.00 | 22/08/2024 | 20:00 | 23/08/2024 | 00:00 | **Yes** |
|  | LFI thorbjorn2 | 1080p | 25.00 | 22/08/2024 | 20:00 | 23/08/2024 | 23:00 | **Yes** |
|  | LFI vogar | 1080p | 25.00 | 22/08/2024 | 20:00 | 04/09/2024 | 00:00 | Facing wrong way |
|  | MBL webcam1 | 1080p | 25.00 | 22/08/2024 | 21:00 | 29/08/2024 | 23:00 | **Yes** |
|  | MBL webcam11 | 1080p | 25.00 | 22/08/2024 | 21:00 | 25/08/2024 | 23:00 | **Yes** |
|  | MBL webcam13 | 1080p | 24.88 | 22/08/2024 | 20:00 | 22/08/2024 | 23:00 | Facing wrong way |
| *Total hours of footage: 2286* | | | | | | | | |
| ***Table.S5:*** *The webcam footage available for each of the eruptions and the duration of the eruption they covered. Cameras that were used for analysis have been identified and reasons other cameras were not used stated. Most cameras were disregarded for facing the wrong way, not being correctly zoomed or having poor camera quality. LFI Langihruggur is situated on a ridge to the east of the lava flows; all other used webcams are on Þorbjörn facing different directions during eruptions. Webcam footage has been provided by the Icelandic Met Office (thanks to M. Parks and G. Pedersen).* | | | | | | | | |

Table.S6: Fissure segment opening times

| ***Fissure*** | ***Opening time*** | ***Propagation ends north*** | ***Propagation ends south*** | ***Propagation ends total*** |
| --- | --- | --- | --- | --- |
| **December 2023 Eruption** | | | | |
| F1a | 22:58:21 |  |  |  |
| F1b | 22:37:10 |  |  |  |
| F1c | 22:26:34 |  |  |  |
| F2a | 22:22:23 | 22:26:48 |  | 22:26:48 |
| F2b | 22:17:18 |  | 22:26:07 | 22:26:07 |
| F3a | 22:27:32 | 22:31:49 |  | 22:31:49 |
| F3b | 22:26:07 | 22:26:37 | 22:29:09 | 22:29:09 |
| F4a | 22:33:23 | 22:34:00 |  | 22:34:00 |
| F4b | 22:31:31 | 22:33:06 | 22:32:14 | 22:33:06 |
| F4c | 22:32:02 | 22:32:56 | 22:35:38 | 22:35:38 |
| F5a | 22:36:52 | 22:41:50 | 22:41:50 | 22:41:50 |
| F5b | 22:38:41 |  |  |  |
| F5c | 22:56:42 |  | 23:01:50 | 23:02:01 |
| F6a | 23:00:36 |  |  |  |
| F6b | 23:02:12 |  | 23:05:27 | 23:05:27 |
| F7a | 23:07:29 |  |  | 23:10:00 |
| F7b | 23:11:12 |  |  | 23:50:00 |
| F7c | 23:55:31 |  |  |  |
| F7d | 23:59:49 |  |  | 00:12:04 |
| **January 2024 Eruption** | | | | |
| F1a | 08:51:24 |  |  | 09:01:20 |
| F1b | 07:57:06 | 08:14:15 | 08:21:42 | 08:21:48 |
| F1c | 08:06:30 | 08:11:16 | 08:14:30 | 08:14:30 |
| F1d | 08:13:23 | 08:15:03 | 08:14:15 | 08:15:03 |
| F1e | 08:16:00 |  | 08:18:16 | 08:18:16 |
| F2a | 08:18:07 |  | 08:20:00 | 08:20:00 |
| F2b | 08:19:20 |  | 08:28:11 | 08:28:11 |
| F2c | 09:07:55 |  | 09:15:29 | 09:15:29 |
| F3a | 12:09:50 | 12:19:58 |  | 12:19:58 |
| F3b | 12:06:07 |  |  | 12:10:33 |
| F3c | 12:32:24 |  | 12:37:01 | 12:37:01 |
| F3d | 12:50:19 |  |  | immediately at full extent |
| F3e | 13:01:13 |  |  | immediately at full extent |
| **February 2024 Eruption** | | | | |
| 1a | 06:32:59 | 06:39:15 |  | 06:39:15 |
| 1b | 06:31:15 |  |  | 06:31:15 |
| 2 | 06:24:07 |  |  | 06:24:07 |
| 3 | 06:02:03 |  |  | 06:02:03 |
| 4a | 06:06:21 | 06:06:31 |  | 06:06:31 |
| 4b | 06:06:41 |  |  | 06:06:41 |
| 4c | 06:17:23 |  | 06:19:54 | 06:19:54 |
| 4d | 06:22:56 | 06:24:26 | 06:29:52 | 06:29:52 |
| 4e | 06:35:31 | 06:36:09 |  | 06:36:09 |
| 4f | 06:39:22 |  |  | 06:39:22 |
| 5a | 06:42:13 |  |  | 06:42:13 |
| 5b | 06:42:19 | 06:44:29 | 06:44:57 | 06:44:57 |
| **March 2024 Eruption** | | | | |
| 2a | 20:27:19 | 20:33:55 |  | 20:33:55 |
| 2b | 20:23:52 | 20:26:54 | 20:24:12 | 20:26:54 |
| 2c | 20:22:58 | 20:23:59 | 20:24:08 | 20:24:08 |
| 2d | 20:24:44 |  | 20:25:18 | 20:25:18 |
| 2e | 20:26:04 | 20:26:16 | 20:29:42 | 20:29:42 |
| 2f | 20:28:12 | 20:29:42 | 20:30:37 | 20:30:37 |
| 3a | 20:32:46 | 20:33:34 | 20:37:56 | 20:37:56 |
| 3b | 20:38:16 |  |  | 20:38:16 |
| 3c | 20:49:35 | 20:50:52 |  | 20:50:52 |
| 3d | 20:52:14 | 21:01:47 |  | 21:01:47 |
| 4 | 21:05:40 | 21:06:14 | 21:08:11 | 21:08:11 |
| **May 2024 Eruption** | | | | |
| 1a | 13:01:30 | 13:07:55 |  | 13:07:55 |
| 1b | 12:56:06 | 12:59:29 |  | 12:59:29 |
| 1c | 12:51:12 | 12:54:06 |  | 12:54:06 |
| 1d | 12:48:30 | 12:50:03 |  | 12:50:03 |
| 1e | 12:46:59 | 12:48:06 | 12:48:30 | 12:48:30 |
| 1f | 12:46:12 | 12:47:13 | 12:51:12 | 12:51:12 |
| 2a | 12:52:57 | 12:53:26 | 12:53:14 | 12:53:26 |
| 2b | 12:55:46 |  |  | 12:55:46 |
| 3a | 12:56:03 |  | 12:59:52 | 12:59:52 |
| 3b | 13:01:13 |  | 13:26:42 | 13:26:42 |
| 3c | 13:30:45 |  |  | 13:30:45 |
| 3d | 13:51:09 | 13:54:42 |  | 13:54:42 |
| 3e | 13:47:02 | 13:48:09 | 13:49:20 | 13:49:20 |
| 3f | 13:57:20 |  | 14:21:57 | 14:21:57 |
| 3g | 15:51:40 |  | 16:36:35 | 16:36:35 |
| **August 2024 Eruption** | | | | |
| 1 | 03:50:00 | 05:30:00 |  | 05:30:00 |
| 2a | 02:25:00 | 02:38:00 |  | 02:38:00 |
| 2b | 02:12:00 |  |  |  |
| 2c | 02:12:00 |  |  |  |
| 2d | 02:12:00 |  |  |  |
| 3ab | 22:30:42 | 22:46:08 |  | 22:46:08 |
| 3c | 21:43:04 | 22:23:59 |  | 22:23:59 |
| 4a | 21:25:50 | 21:42:29 | 21:26:08 | 21:42:29 |
| 4b | 21:27:16 | 21:27:54 | 21:34:15 | 21:34:15 |
| 5a | 21:34:57 |  | 21:43:08 | 21:43:08 |
| 5b | 21:44:42 | 22:01:00 |  | 22:01:00 |
| 5c | 22:01:04 |  |  |  |
| 5d | 22:08:44 |  |  |  |
| ***Table.S6:*** *Fissure segment propagation initiation times, and times of complete fissure segment propagation. Some fissure segments are missing some propagation times because of obstructions in the webcam footage or footage resolution limits.* | | | | |

Fig.S1: Fissure segment labels


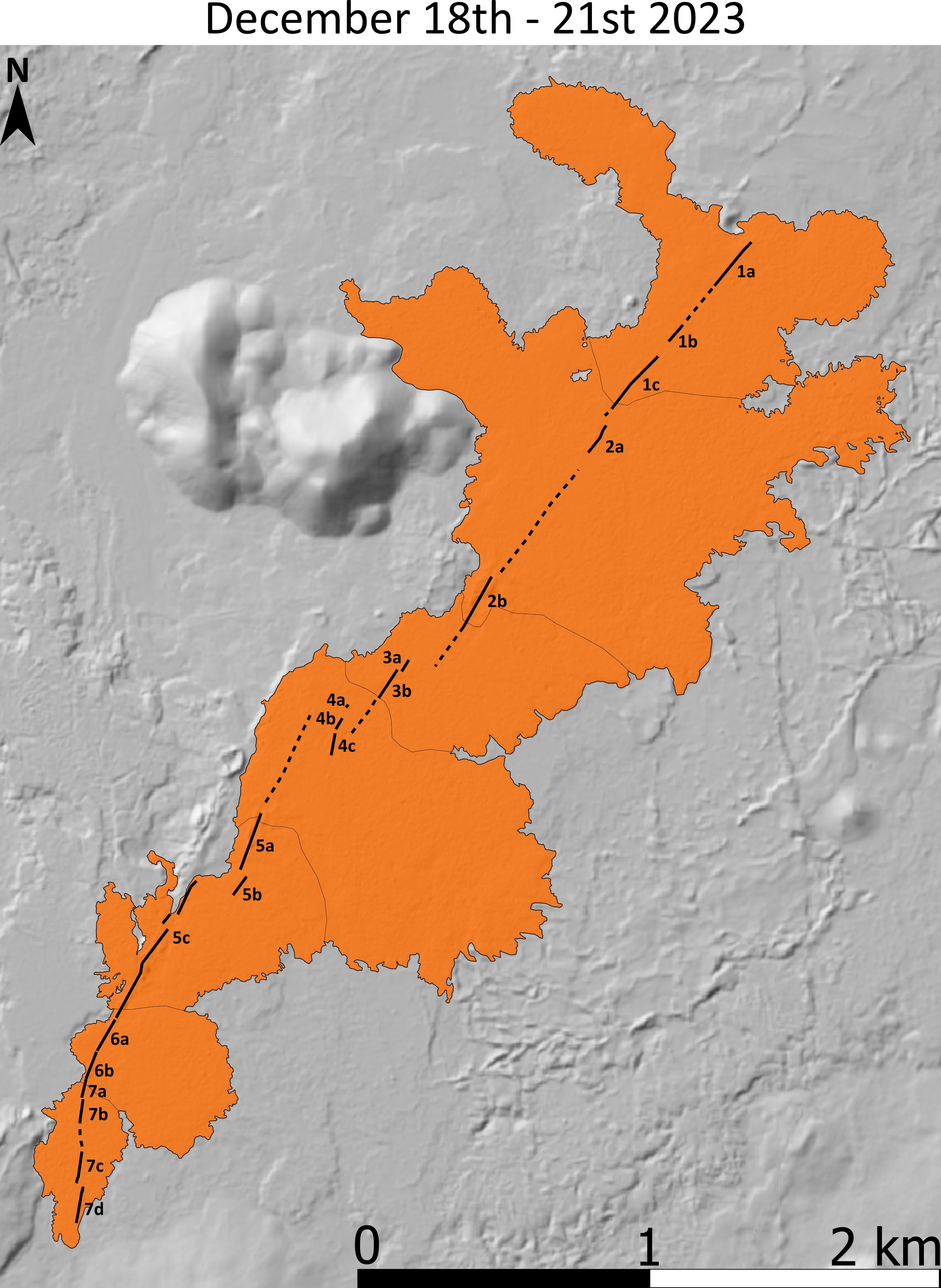

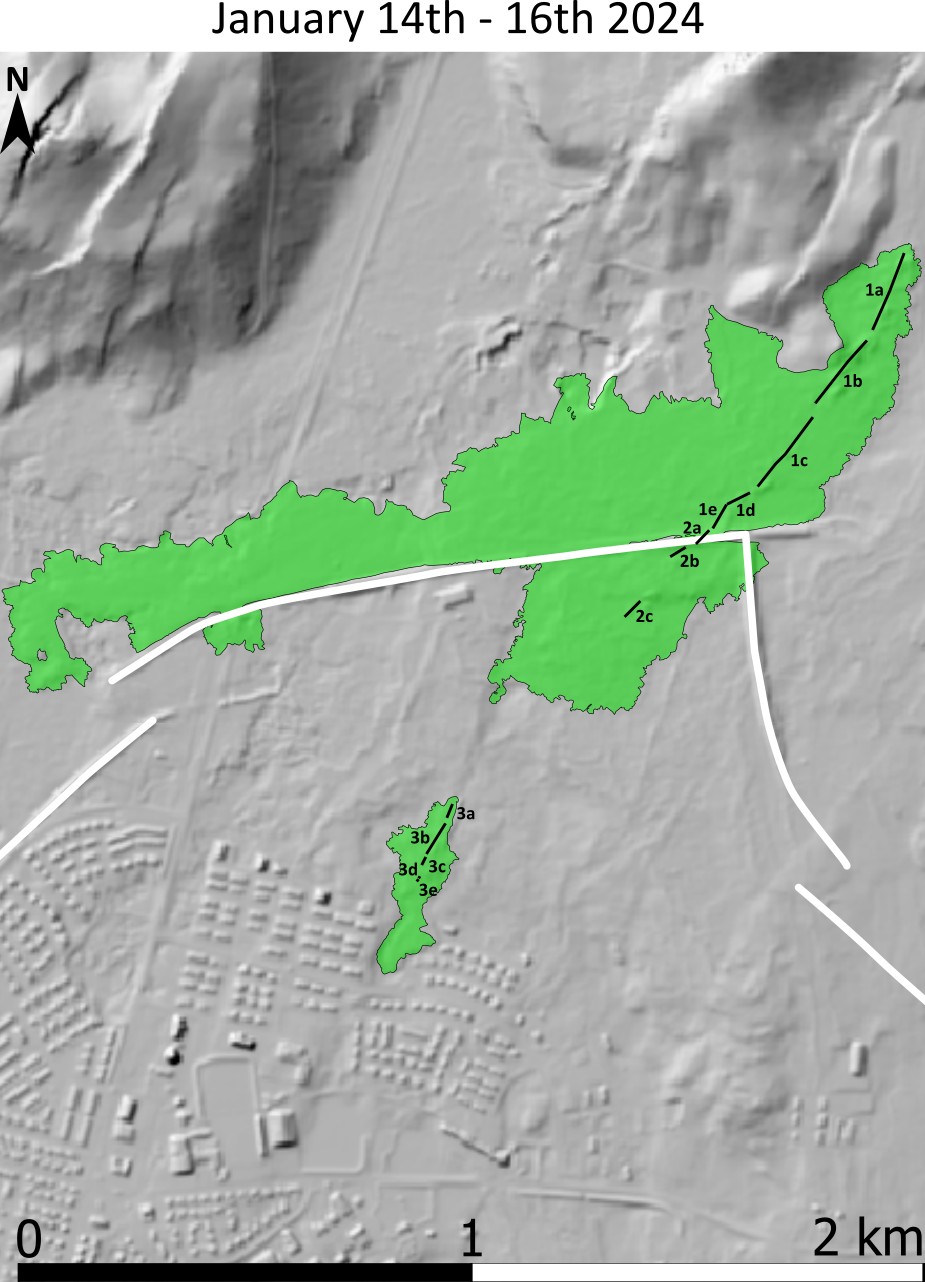

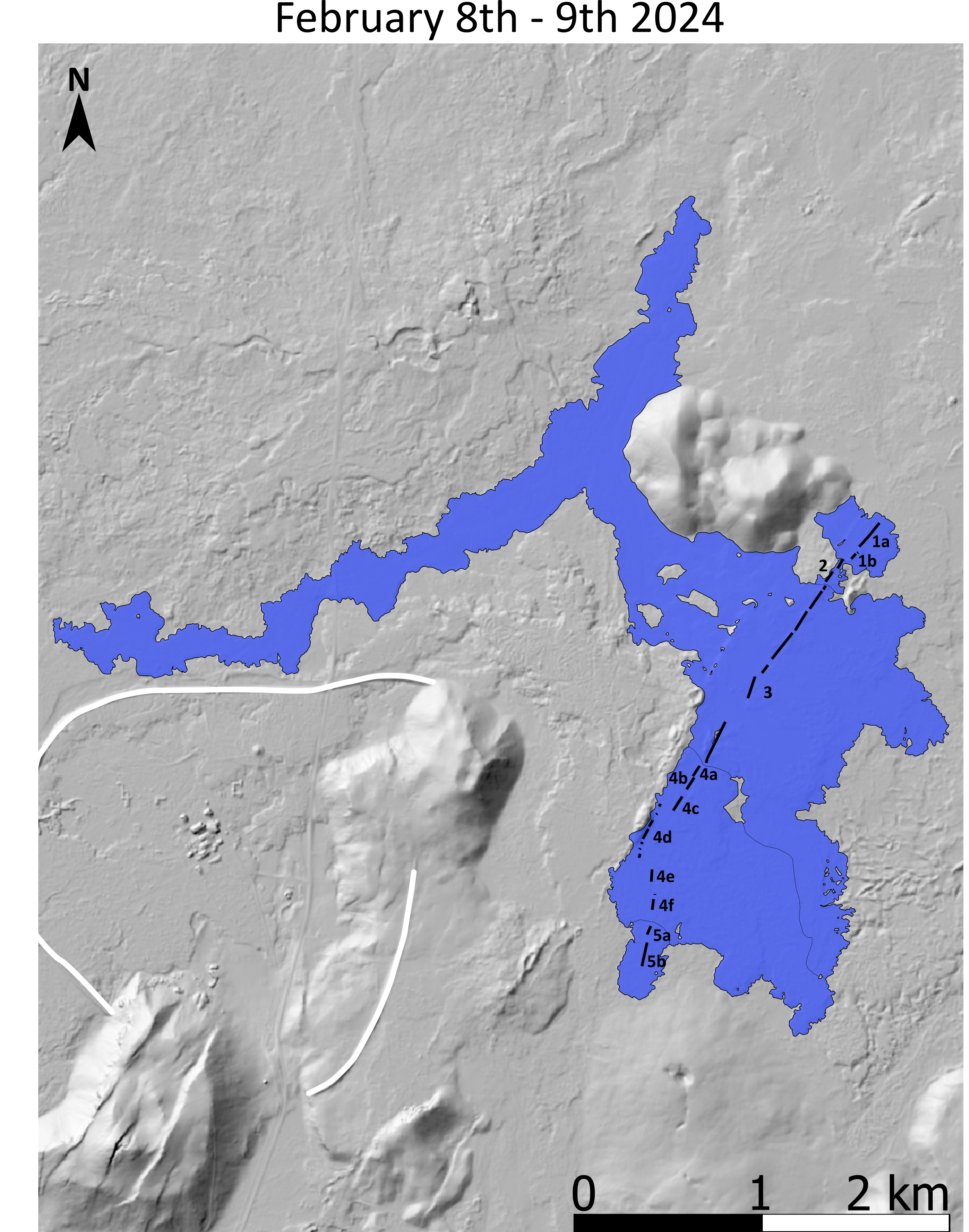

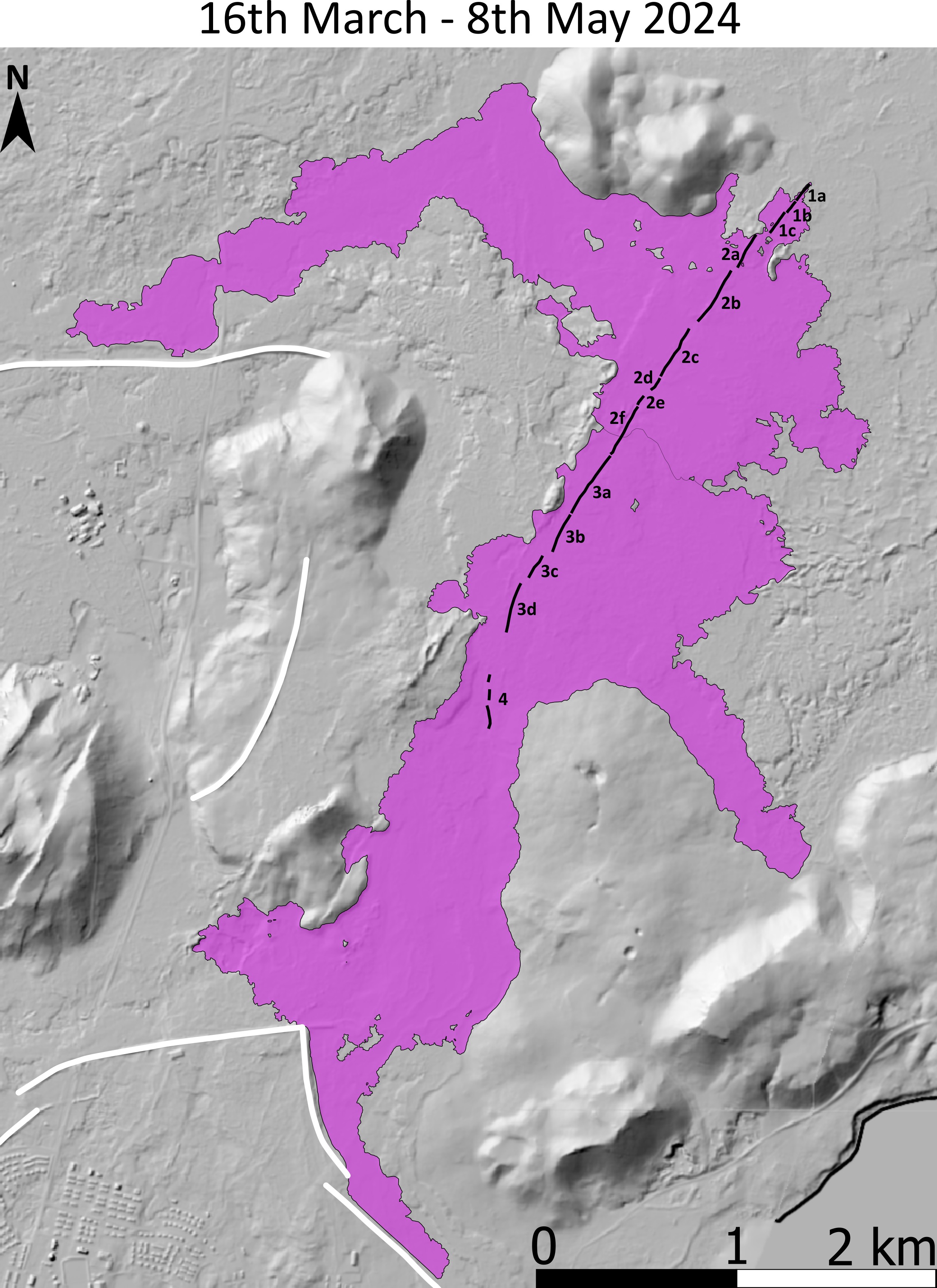

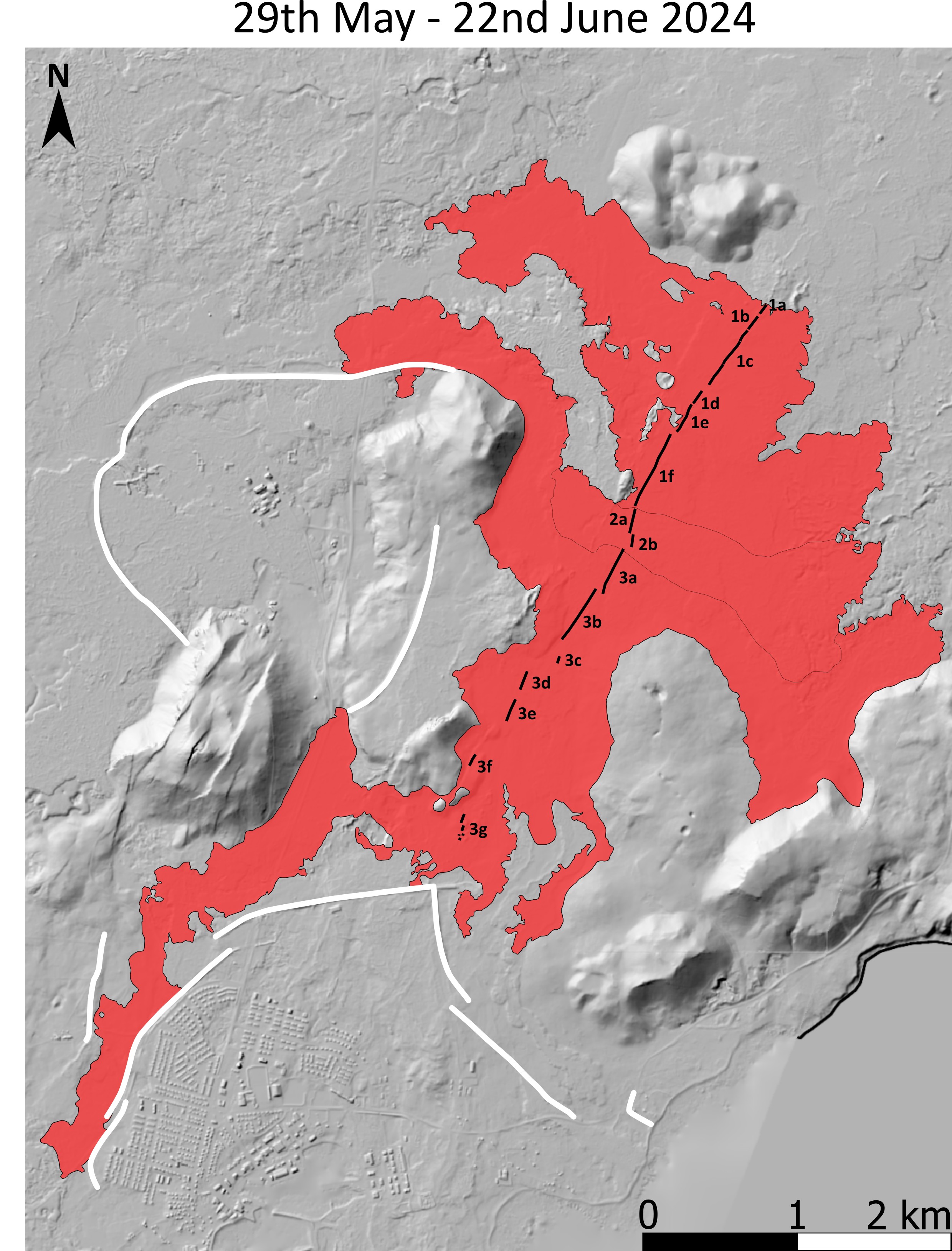


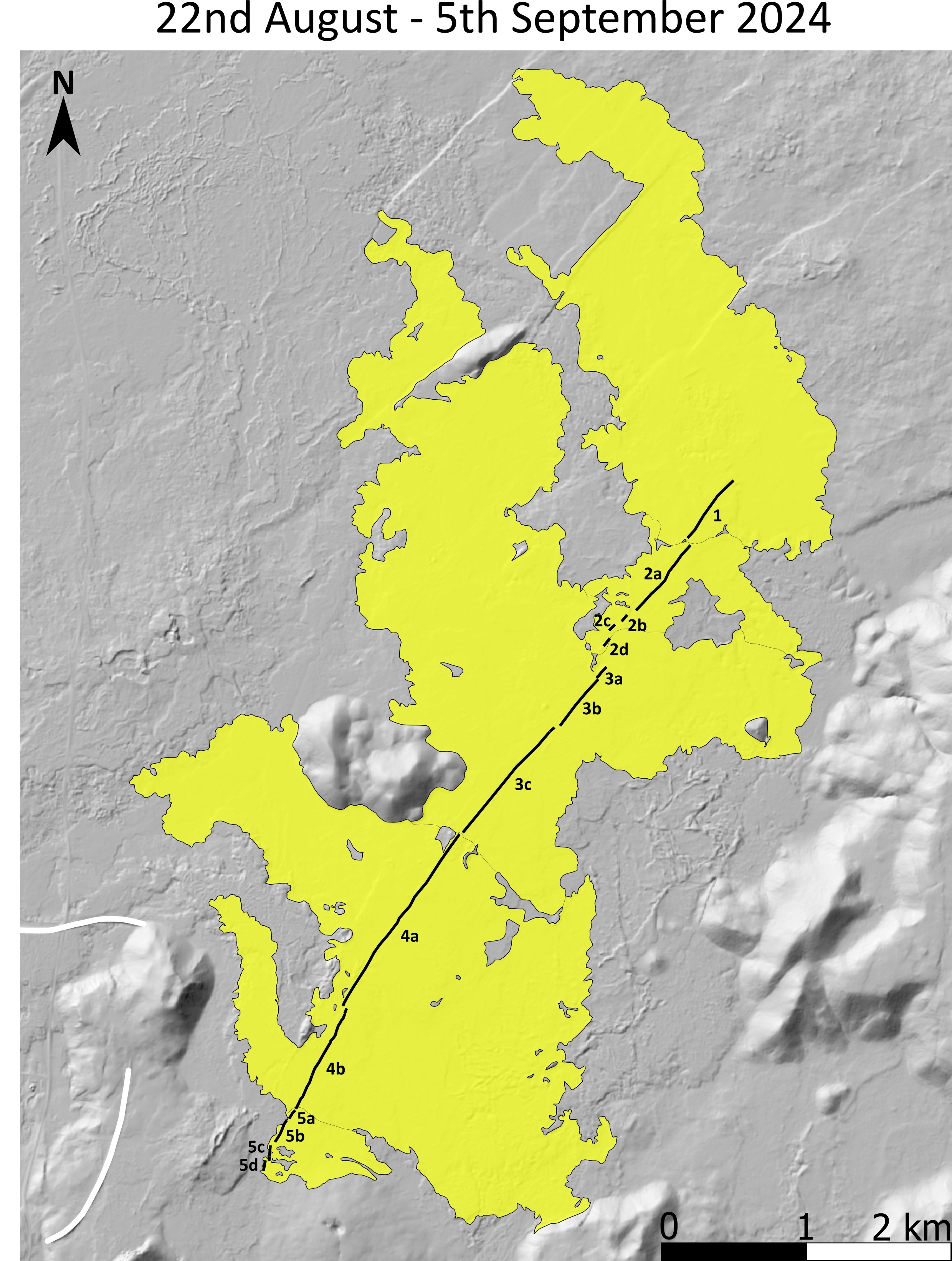


Fig.S2: Ruler uncertainty explanation


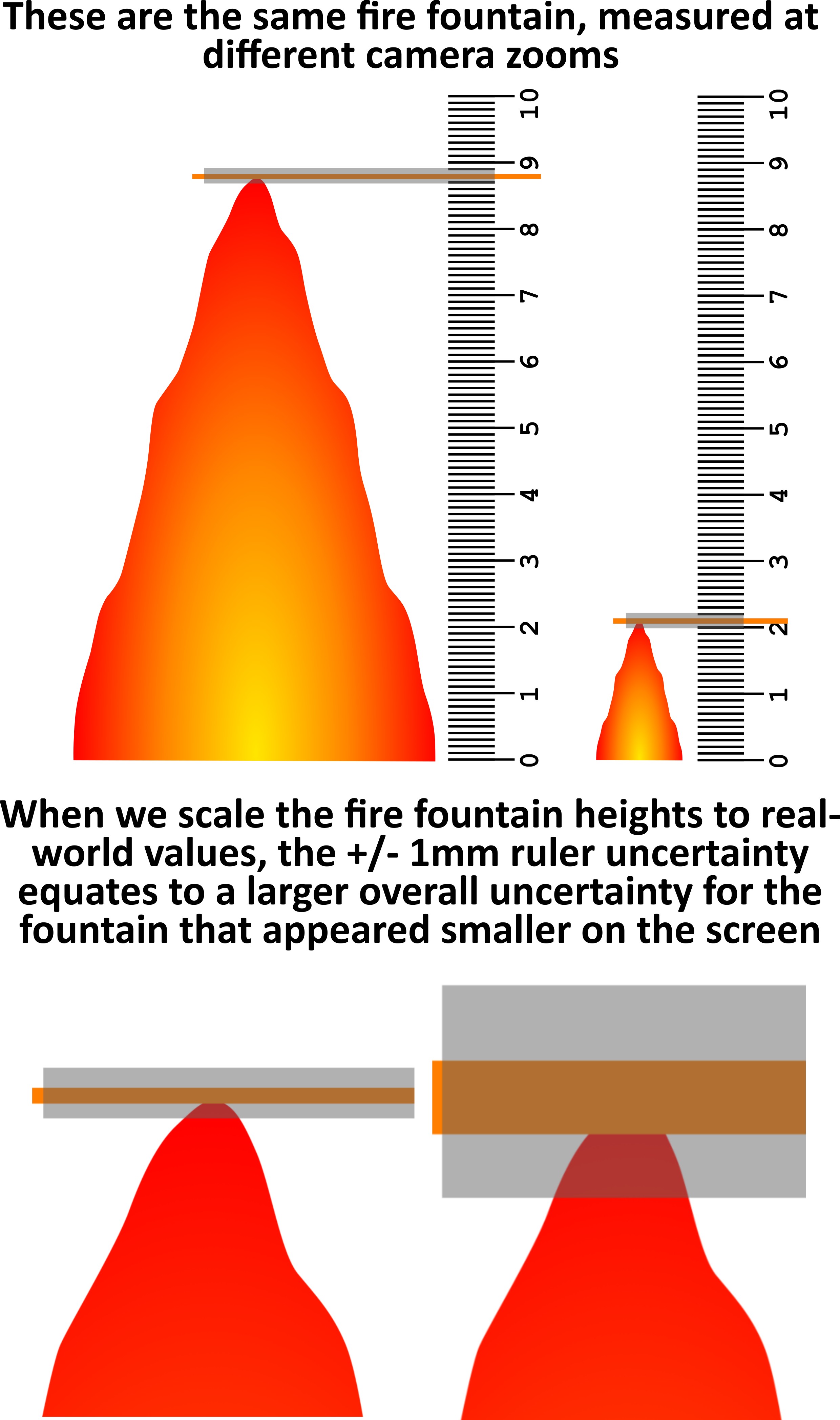


If we view the same fire-fountain, with the same height, when the footage is zoomed further out, the fire-fountain appears much smaller on the screen. A higher scaling factor must then be used to turn the screen height into the real-world height. The scaling factor is also applied to the +/-1mm ruler uncertainty, meaning the uncertainty is stretched significantly when we scale to real-world heights. When the camera is zoomed in closely, the fire-fountain appears larger on the screen. A smaller scaling factor must then be used to scale the fire-fountain to a real-world height. This means we are stretching the ruler uncertainty by less, and the resultant uncertainty on the measurement is smaller.

Fig.S3: Angular uncertainty explanation


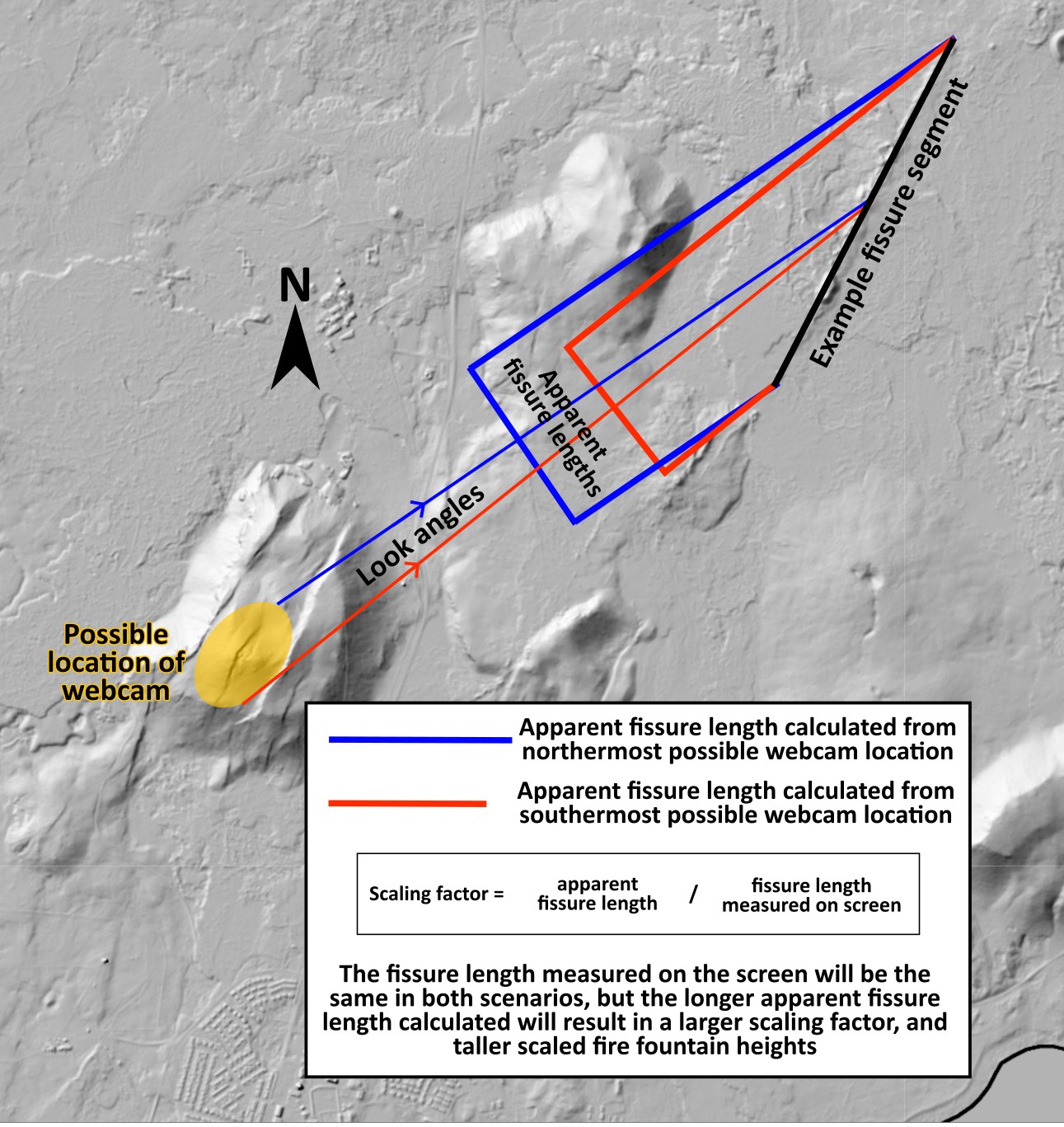


The webcams have been moved between eruptions, and a good record has not been kept of the exact location of each webcam during each eruption. From surrounding features seen on the footage and the fact that the camera needs to be placed on high, flat ground, a small area has been marked as possible locations of the cameras. The look angle of the camera affects the apparent fissure length seen in the footage, which affects the scaling factor. Apparent fissure length calculations have been made from the northernmost point in the area and the southernmost point in the area, and their results applied to the scaling factor to get the uncertainty from viewing angle uncertainty. The more oblique the fissure is to the camera, the greater the effect that this unknown viewing angle will have on the heights of scaled fire-fountains.

Figure.S4: Measurement and scaling protocol for fire-fountains


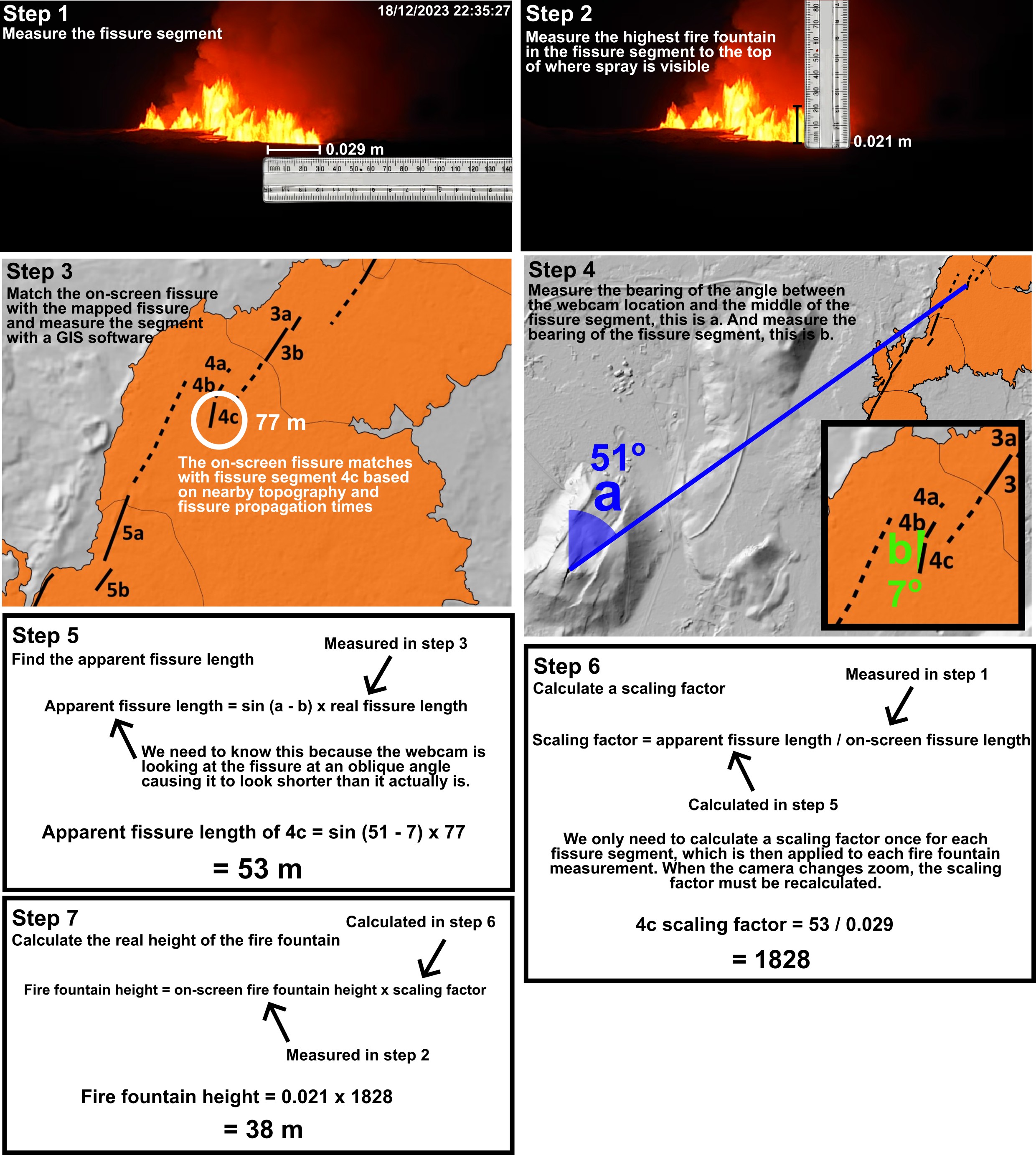


Measurement and scaling protocol for fire-fountains, measuring and scaling on-screen measurements.

1. Measure the fissure segment on-screen that the fire-fountain originates. The fissure segments here were identified based on propagation patterns and breaks seen in the curtain of fire.
2. Measure the highest fire-fountain in the fissure segment from the top of where the spray is visible. If there is more zoomed-in footage, it is helpful to look at that first to better identify how far up the lava spray is going to work out how much of the diffuse cloud is lava spray. In this study, all of the diffuse clouds were identified as lava spray. It is important to keep your definition of the top of the fire-fountain the same throughout all the observations.
3. Match the onscreen fissure segment to the map using the method outlined in Fig.4.
4. Measure the bearing of the angle between the webcam location and the middle of the fissure segment, and the bearing of the fissure segment.
5. Find the apparent fissure length at real-world scale
6. Calculate the scaling factor
7. Use the scaling factor to scale your fire-fountain measurements to real-world heights.
